# Antitumoral Efficacy of AuNRs‐Laden ECFCs In Vitro and In Vivo: Decoding the Heat and Rays Combo Treatment in Breast Cancer and Melanoma Cells

**DOI:** 10.1002/adhm.202502416

**Published:** 2025-06-23

**Authors:** Cecilia Anceschi, Francesca Scavone, Paolo Armanetti, Luca Menichetti, Claudia Catarinicchia, Claudia Borri, Fulvio Ratto, Filippo Micheletti, Noemi Formica, Jessica Ruzzolini, Elena Frediani, Anastasia Chillà, Francesca Margheri, Mirko Severi, Rita Traversi, Patrizia Nardini, Daniele Guasti, Mario Del Rosso, Tommaso Del Rosso, Lisa Giovanelli, Cinzia Talamonti, Monica Mangoni, Isacco Desideri, Silvia Burchielli, Fabiola Pajar, Gabriella Fibbi, Anna Laurenzana

**Affiliations:** ^1^ Department of Experimental and Clinical Biomedical Sciences University of Florence Viale Morgagni 50 Florence 50134 Italy; ^2^ Institute of Clinical Physiology (IFC) National Research Council Pisa 56124 Italy; ^3^ Institute of Applied Physics “N. Carrara” National Research Council Sesto Fiorentino 50019 Italy; ^4^ Department of Chemistry “Ugo Schiff,” University of Florence Sesto Fiorentino 50019 Italy; ^5^ Department of Experimental and Clinical Medicine University of Florence Viale Pieraccini 6 Florence 50134 Italy; ^6^ Department of Physics Pontifical Catholic University of Rio de Janeiro Rua Marquês de São Vicente 225, Gávea Rio de Janeiro 22451–900 Brazil; ^7^ Department of Neurosciences Psychology Drug and Child Health Area (NEUROFARBA) University of Florence Viale Pieraccini 6 Florence 50134 Italy; ^8^ Center for Experimental Biomedicine Research Area of CNR Pisa 50134 Italy; ^9^ Department of Translational Research and New Technologies, St. Chiara Hospital University of Pisa Via Savi, 10 Pisa 56100 Italy

**Keywords:** autophagy, breast cancer, Endothelial Colony Forming Cells (ECFCs), ferroptosis, gold nanorods, hyperthermia, melanoma, radiotherapy

## Abstract

Radiotherapy remains a cornerstone in metastatic cancer treatment but is often hindered by tumor hypoxia and radioresistance. Gold nanorods (AuNRs) offer promise in enhancing radiotherapy through hyperthermia, yet their clinical impact is limited by poor tumor targeting. Building on the previous findings demonstrating the tumor‐homing ability of Endothelial Colony Forming Cells (ECFCs) loaded with AuNRs, this study advances their use as a biologically targeted delivery system for precise radiotherapy enhancement. Using 3D in vitro tumor models and in vivo studies with nude rats, it is demonstrated that ECFCs actively home to hypoxic tumor regions, overcoming traditional nanoparticle delivery limitations. This targeted approach ensures efficient AuNR accumulation, enhancing photothermal activation and maximizing radiosensitization. In vitro, ECFC‐loaded AuNRs significantly amplify radiotherapy effects, inducing ferroptosis in melanoma and inhibiting autophagy in breast cancer cells—revealing distinct tumor‐specific mechanisms. Moreover, ECFC‐AuNRs suppress tumor proliferation and angiogenesis, blocking vessel‐like structure formation in vitro and in vivo. By integrating cellular therapy with nanotechnology, this study presents a novel strategy to counter radioresistance and improve therapeutic precision. These findings lay the foundation for a clinically viable, patient‐specific approach, unlocking new possibilities in advanced cancer treatment.

## Graphical abstract

Endothelial Colony Forming Cells (ECFCs) acted as cellular cyborgs, stealthily transporting gold nanorods (AuNRs) into tumors to enable targeted near‐infrared (NIR) hyperthermia combined with radiotherapy. This approach triggers ferroptosis in melanoma and inhibits autophagy in breast cancer, revealing a tumor‐specific response to nanomaterial‐assisted combination therapy.

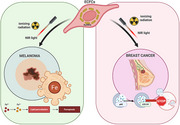

## 1 Introduction

In the landscape of cancer treatment, radiotherapy has long stood as a cornerstone, employed as a standard and essential approach for various types of metastatic cancers.^[^ ^1^, ^2^, ^3^ ^]^ Its therapeutic impact on cancer cells and tumor tissue is achieved through a dual mechanism, encompassing both direct and indirect pathways^[^ ^4^, ^5^ ^]^ In the direct impact pathway, radiation induces single‐strand breaks (SSB) and double‐strand breaks (DSB) in DNA, consequently halting cell division and proliferation and potentially leading to cell necrosis and death.^[^ ^6^ ^]^ Conversely, the indirect action of radiotherapy involves the generation of reactive oxygen species (ROS).^[^ ^7^, ^8^ ^]^ These ROS play a pivotal role by causing damage to biomolecules, inducing cellular stress, and significantly altering signaling pathways within cells.^[^ ^6^ ^]^ Currently, daily fractions of 1.5 to 3 Gray(Gy) of ionizing radiation are typically administered over several weeks.^[^ ^9^, ^10^, ^11^, ^12^ ^]^ However, the response to radiation varies across specific cancers, with some, like lymphomas and seminomas, exhibiting high sensitivity to modest doses, while others, such as melanoma and glioblastoma, prove highly radioresistant even after extensive radiation exposure.^[^ ^13^, ^14^, ^15^, ^16^ ^]^ Despite its efficacy, several challenges persist in effectively employing radiation to treat metastatic tumors.^[^ ^17^ ^]^ These challenges include the presence of cancer stem cells, tumor heterogeneity, angiogenesis, vasculogenic and metabolic changes. Notably, hypoxia in the tumor microenvironment emerges as a significant hurdle. In contrast to the normal oxygen microenvironment, tumor cells display substantial radiation resistance in hypoxic conditions.^[^ ^18^, ^19^, ^20^, ^21^ ^]^ Indeed, the decreased availability of oxygen diminishes the oxygen enhancement effect, which is crucial for the positive impact of ionizing radiation. Moreover, in the presence of hypoxia, cancer cells become more aggressive,^[^ ^22^ ^]^ by activating survival pathways, such as the hypoxia‐inducible factor (HIF) pathway. HIF enhances cell survival in low‐oxygen conditions by activating genes that drive angiogenesis, metabolic adaptation, resistance to apoptosis, and metastatic pathways.^[^ ^23^, ^24^, ^25^, ^26^ ^]^ These hurdles necessitate innovative approaches to enhance the precision and effectiveness of radiotherapy.^[^ ^27^ ^]^ Nanomedicine,^[^ ^28^, ^29^ ^]^ especially the utilization of gold nanoparticles (AuNPs), presents a promising avenue to overcome these challenges. AuNPs, owing to their high atomic number and biocompatibility, act as effective radiosensitizers.^[^ ^30^ ^]^ Their ability to absorb, scatter, and emit radiation energy, coupled with the generation of reactive oxygen species, enhances the radiosensitivity of tumor cells.^[^ ^30^, ^31^, ^32^ ^]^ Additionally, a particularly intriguing aspect lies in the unique properties of anisotropic AuNPs, such as gold nanorods AuNRs, which exhibit plasmonic oscillations in the near‐infrared window (NIR, 700–1100 nm) of minimal biomolecule interaction and maximal biotissue penetration. This feature allows them to selectively absorb and mediate the conversion of NIR light into heat, and thus to raise the temperature of their environment beyond the critical threshold of 43 °C for hyperthermia applications.^[^ ^33^, ^34^ ^]^ By utilizing the photothermal properties of AuNPs, it is feasible to precisely regulate and elevate temperatures within specific tissues based on their distribution and extraction characteristics. This precise hyperthermia enables targeted disease treatment, such as cancer, where elevated temperatures selectively damage cancer cells with minimal side effects. Unfortunately, numerous obstacles persist in the effective utilization of NPs in cancer therapy. Despite active targeting with tumor‐binding units, a significant portion of administered NPs inevitably accumulates in healthy organs and tissues, thereby reducing treatment efficacy.^[^ ^35^, ^36^, ^37^ ^]^ This inefficiency, reflected in less than 5% of the injected dose reaching the tumor, is exacerbated by the challenging microenvironment within solid tumors. Elevated interstitial fluid pressure and high solid stress characterize this hostile milieu, collectively contributing to the suboptimal intra‐tumoral distribution of NPs.^[^ ^35^, ^36^, ^37^ ^]^

The unique capability of specific stem, progenitor, and immune cells, termed cytotherapeutics, to target tumors has paved the way for their application as vectors in delivering various anticancer payloads.^[^ ^34^, ^38^, ^39^, ^40^, ^41^, ^42^, ^43^ ^]^ In contrast to nanoparticles, which rely on passive concentration gradients, cells engage in an active targeting process, allowing them to infiltrate the tumor interstitium. This active process enables cells to overcome stromal barriers and the elevated interstitial pressure typical in most tumors, facilitating their access to tumor sites. This not only optimizes the therapeutic payload delivered to cancerous tissues but also minimizes off‐target effects, potentially revolutionizing the landscape of cancer treatment. In our study, we leverage Endothelial Colony Forming Cells, (ECFCs), a subtype of Endothelial Progenitor Cells (EPCs) for the targeted delivery of AuNRs to cancer tissue. Building upon our previous research, which proved the successful migration of ECFCs loaded with AuNPs to tumor masses upon administration through the mice tail vein,^[^ ^39^ ^]^ our current study delves into a novel exploration.

The paramount objective of this study is to underscore the translational significance of these cargo cells and elucidate their distinct advantages over conventional tumor treatments that rely solely on gold nanoparticles. Our study reveals that ECFCs exhibit superior uptake of AuNRs, translating into heightened therapeutic properties, including amplified photoacoustic signals and enhanced photothermal responses. These findings position ECFCs loaded with AuNRs as potent candidates for advancing cancer therapy beyond the limitations of conventional treatments. Furthermore, by employing 3D tumor models and conducting in vivo studies utilizing nude rats, we assessed the antitumoral activity displayed by these cargo cells. Our findings reveal that ECFC loaded with AuNRs not only effectively restrained tumor cell growth and dissemination but also inhibited the intrinsic capability of tumor cells to form vessel‐like structures. Most importantly our investigation extended to in vivo studies with nude rats, reveals the capacity of ECFCs laden with AuNRs to selectively home in hypoxic zones within the tumor microenvironment. In this milieu, we propose a novel approach that combines AuNRs‐based hyperthermia with radiotherapy to address the challenge of radioresistance. This synergistic strategy aims to unlock new dimensions in cancer treatment, leveraging the unique strengths of both modalities to overcome the limitations posed by radioresistant tumors. Indeed, we assessed not only the effectiveness of the combo‐treatment (radiotherapy and hyperthermia) in melanoma and breast cancer, but we also successfully unveiled the mechanism underlying the cooperative effects. Notably, the combined treatment induced cell damage in a unique, cancer‐specific manner for each cancer type. Specifically, we found that the dual treatment triggered ferroptosis^[^ ^44^, ^45^, ^46^, ^47^ ^]^ in melanoma cells, offering a novel avenue for inducing programmed cell death in this particular cancer type. In breast cancer cells, the treatment effectively suppressed autophagy—a critical cellular process linked to the development of radioresistance and chemoresistance. Our ability to delineate these specific cellular responses marks a significant stride in understanding the interplay between hyperthermia and radiotherapy. The induction of ferroptosis in melanoma cells and the suppression of autophagy in breast cancer cells provide valuable insights into potential tailored therapeutic strategies.^[^ ^45^, ^48^ ^]^ These findings not only contribute to advancing our comprehension of the cooperative effects in cancer treatment guided by the unique attributes of ECFCs but also pave the way for targeted interventions, offering a personalized approach to effectively address melanoma and breast cancer.

## 2 Results

### 2.1 Enhanced Photoacoustic and Photothermal Characteristics Through Gold Nanorods Enrichment

**Figure 1:**
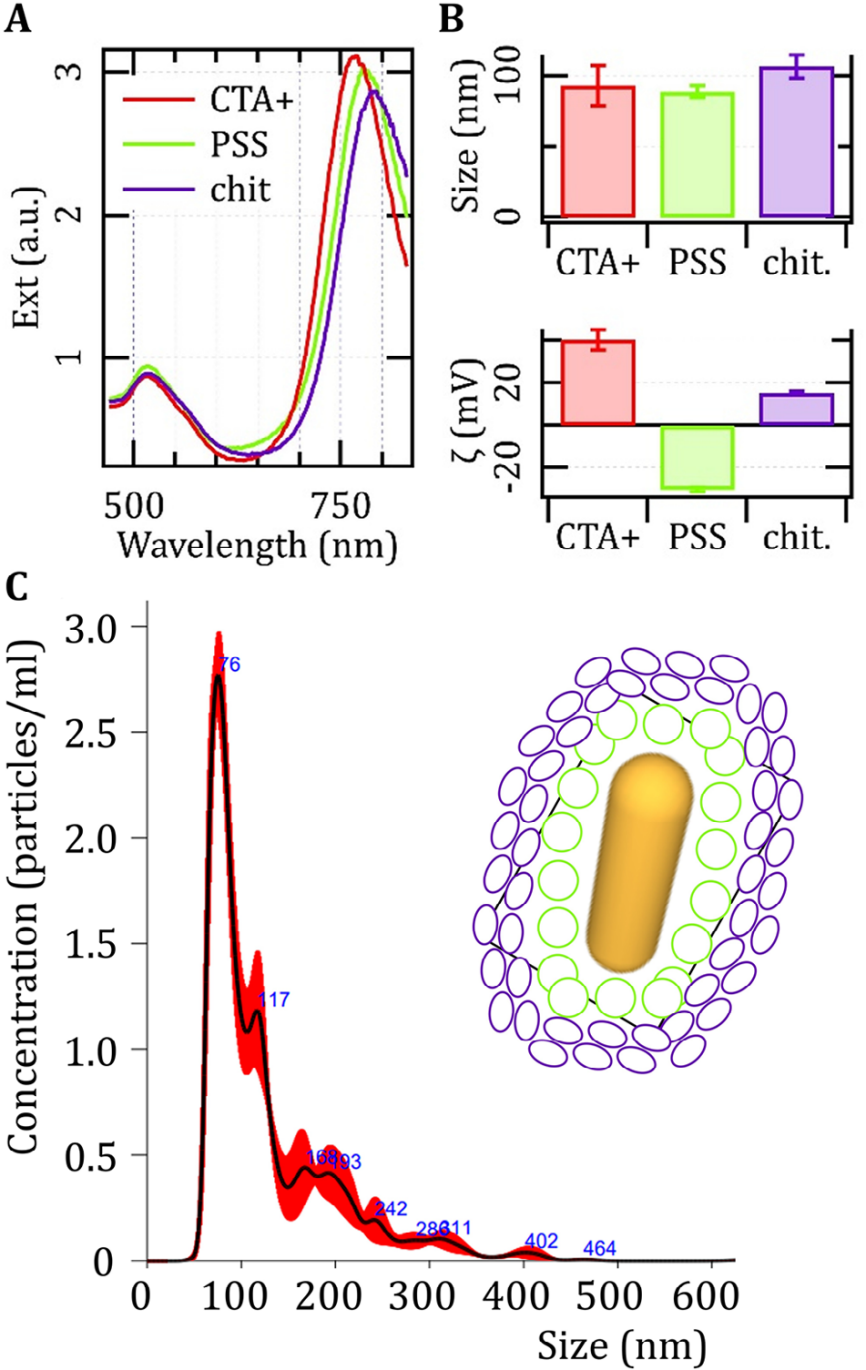
Chemical and physical characterization of gold Nanorods (AuNRs). A) Optical extinction spectra and B) Dynamic Light Scattering (DLS) analysis of gold nanorods through various steps of the synthetic protocol from the original CTA+ to the final chitosan coating. C) extract of a NanoSight histogram of the final particles dispersed in buffer confirming the corresponding DLS data and suggesting the presence of small aggregates. The inset provides a schematic representation of a particle coated with PSS (green) and chitosan (blue).

In our previous work,^[^ ^39^ ^]^ as a photothermal trigger, we implemented a so‐called gold nano‐mix, characterized by a heterogeneity of shapes and including in particular a population of plate‐like particles responsible for their optical absorbance in the first biological window. Here, we tested the replacement of the gold nano‐mix with an AuNR (**Figure** **1**) alternative with a so‐called longitudinal mode of plasmonic oscillations ≈780 nm, and a better translational profile, in terms of quality and standardization. However, in an effort to ensure continuity to our previous results, we made sure to coat the AuNRs with the same choice of low molecular weight unmodified chitosan as a cationic polysaccharide, which we also used in the case of the gold nano‐mix, and through the same protocol of electrostatic adsorption. However, unlike the gold nano‐mix, the as‐synthesized AuNRs exhibit a cationic rather than anionic electrokinetic potential due to the use of cetrimonium (CTA+) as a shape‐directing agent and surfactant. Therefore, prior to chitosan adsorption, we replaced the initial surfactant with an anionic polymer commonly used in layer‐by‐layer coatings of biomedical relevance such as sulfonated polystyrene (PSS). Figure 1 compares the spectra of optical extinction (A), hydrodynamic diameters, and electrokinetic potentials (B) of AuNRs across the various steps from CTA+ to PSS and chitosan, which collectively demonstrate their functional modification and colloidal stability.

**Figure 2:**
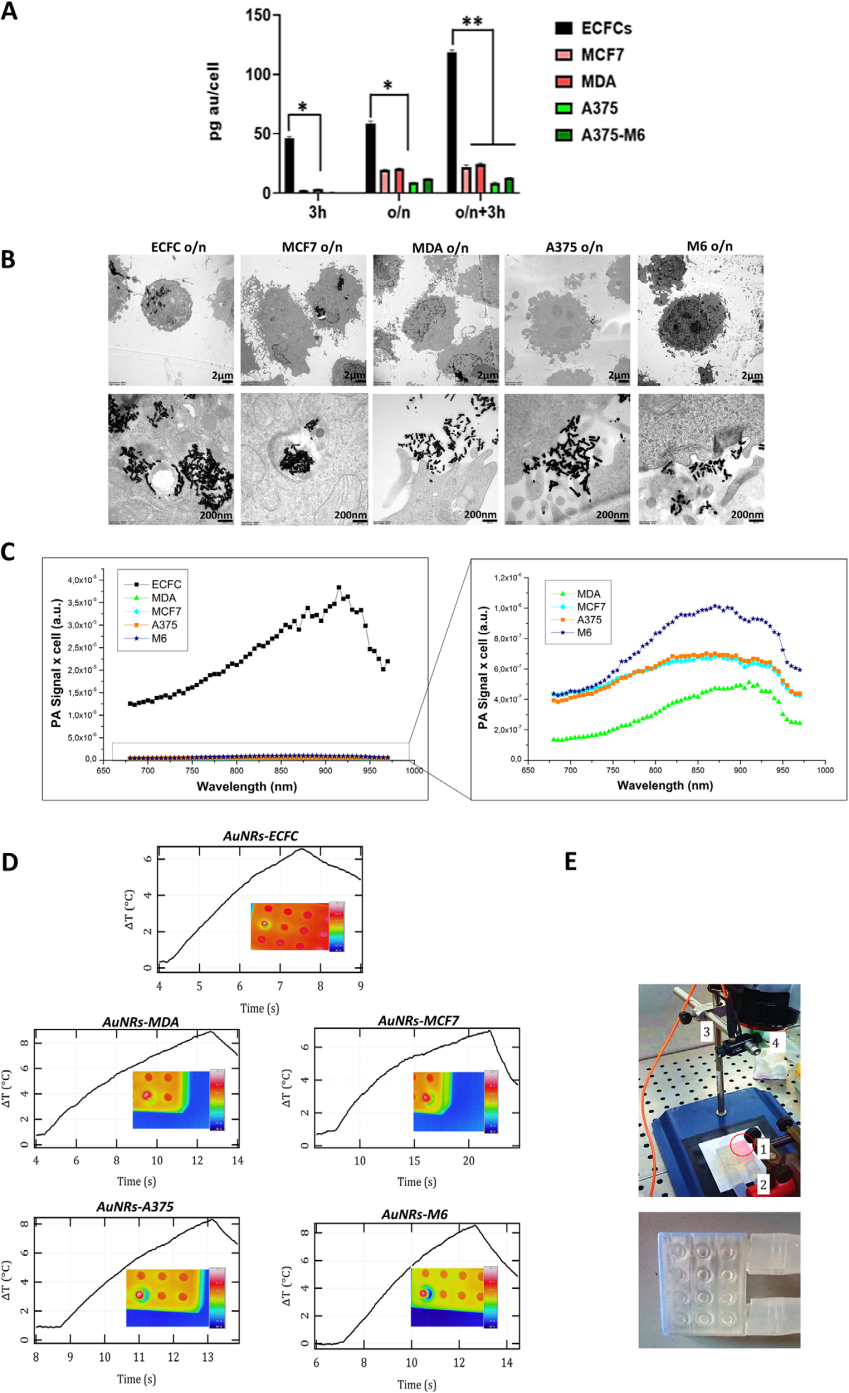
Evaluation of AuNR uptake, localization, and photothermal effects in ECFCs and tumor cells A) ICP analysis of cells treated at different time points with one dose or two consecutive equal doses of AuNPs. Significance was assessed by a one‐way ANOVA test followed by a Newman‐Keuls post‐test. Error bars indicate mean ± SD; asterisks (^*^ *p* < 0.05, ^**^ *p* < 0.001). All experiments were performed independently at least three times. B) Transmission electron micrograph of the indicated cells after overnight treatment with AuNRs. Scale bars represent 2 µm and 200 nm as indicated. C) Plot of the PA spectral trend of AuNP‐ECFC cells in comparison with tumor cells enriched with AuNRs. Enlarged image of the PA spectra of the tumor cells is shown on the right. D) Examples of thermographs recorded after exposing cultures of various cell types treated with chitosan‐coated gold nanorods to 808 nm laser light with a power density of 1 W cm‐2. The inset shows the thermographic images of the temperature detected on the surface of the irradiated well when the co‐culture reached 43 °C. E). Note that the recordings do not begin at the switch‐on event of the laser, which is evident as a change in the slope of the curves. The experimental setup used for photothermal testing, showing an irradiated well 1) in a custom multiwell plate connected to a chiller via silicon tubing 2), and showing a multimode optical fiber 3) and a thermal imaging camera 4). The inset is a photograph of a custom multiwell plate produced by additive manufacturing with a clear resin.

We previously demonstrated that ECFCs robustly internalize chitosan‐coated AuNPs without eliciting cell toxicity.^[^ ^39^ ^]^ In the current study, time‐ dependent uptake of AuNRs was assessed in ECFCs and across four different tumor cell lines: MCF‐7 and MDA‐MB231, representing two breast cancer cell lines, and A375 and A375‐M6, indicative of melanoma. Cells were subjected to a AuNR kinetic treatment before quantifying the cellular uptake using inductively coupled plasma‐atomic emission spectroscopy (ICP‐AES). Cells were treated with AuNRs at the concentrations of 100 × 10^−6^ M Au for 3 h overnight (o/n) and with a double administration of o/n + 3h. **Figure** **2**a shows that the amount of AuNRs internalized by ECFCs increased proportionally with the treatments: ECFCs were capable to increase the intracellular load by fourfold with the single o/n administration, without eliciting toxicity, and by almost tenfold with the double administration (o/n + 3h) compared to the malignant cell lines, showing minimum toxicity (Figure S1, Supporting Information). As no discernible changes were noted in the tumor cell lines following overnight treatment compared to two sequential administrations, the single o/n AuNRs treatment was performed for all further experiments. Transmission electron microscope (TEM) images illustrated the intracellular load with AuNRs in all the above‐mentioned cells (Figure 2B; Figure S2, Supporting Information). The accumulation of AuNRs in ECFCs is significantly higher than in the other cell lines, corroborating the findings from ICP analysis. To explore the potential of ECFCs loaded with AuNRs as theranostic tools, we conducted photoacoustic imaging (PAI) to compare the signal intensity from these loaded cells with that from tumor cells. The results confirmed that the signal intensity from the ECFCs was indeed higher (Figure 2C), further validating the translational potential of using ECFCs as vehicles for AuNRs. PA imaging was performed in vitro using polyethylene (PE) tubes filled with the AuNR enriched cells (ECFCs, MDA, MCF7, A375, M6), then excited by pulsed laser illumination between 680 and 970 nm. The PA signal of cells loaded with AuNRs showed a spectral trend with a maximum intensity ≈900 nm, indicating a redshift of their longitudinal mode of plasmonic oscillations, which is a typical consequence of their endosomal confinement (Figure 2B). The PA signal obtained from the AuNR‐ECFC samples demonstrated a substantial enhancement, nearly 40 times higher than M6, almost 100 times higher than A375 and MCF7, and 200 times higher than MDA (Figure 2C; Figure S3, Supporting Information). This remarkable signal enhancement cannot be solely attributed to the amount of internalized gold; rather, it underscores the distinctive aggregation pattern of gold nanorods within the cells. After exploring the photoacoustic properties, we proceeded to systematically evaluate the efficiency of photothermal conversion across different cell lines. The photothermal properties of AuNRs were tested by measuring the temperature rise of cell suspensions treated with AuNRs (Figure 2D) and then subjected to NIR laser irradiation (1.0 W cm^−2^). The experimental setup involved controlling the base temperature of the cells at their physiological value of 37 °C before performing the NIR laser irradiation in order to reach a range between 43 and 45 °C on the surface of the exposed wells, considered as a regime of mild hyperthermia.^[^ ^49^ ^]^ Our findings indicate a significant increase in temperature within AuNR‐enriched endothelial colony‐forming cells (ECFCs) when compared to other cell lines, specifically M6, A375, MDA, and MCF‐7. The observed temperature elevations varied from 0.2 to 40‐fold (see Figure 2D). This notable result emphasizes the enhanced ability of ECFCs to internalize plasmonic nanoparticles, thereby highlighting their potential as effective candidates for photothermal applications. Cells that were not loaded with gold nanorods and were exposed to NIR light did not detectably heat up.

These findings shed light on the promising potential of ECFCs as carriers for gold nanorods in biomedical applications, specifically in the context of photoacoustic imaging and photothermal hyperthermia. The significant increase in AuNR internalization observed in ECFCs, even with a single overnight administration, underscores the robust nature of these cells in incorporating AuNRs. Importantly, this enhanced uptake was achieved without inducing discernible toxicity, emphasizing the biocompatibility of AuNRs. Furthermore, PAI studies reveal a remarkable signal enhancement in ECFCs loaded with AuNRs compared to pristine tumour cell lines. This distinctive characteristic underscores the potential translational value of ECFCs as effective carriers for AuNRs in theranostic applications. Additionally, the evaluation of photothermal properties indicates that AuNR‐enriched ECFCs exhibit superior efficiency in achieving target temperatures during NIR exposure. This efficiency is particularly critical for potential clinical applications, where sharp thermal gradients are essential for the precise treatment of small metastases.

### 2.2 Tumor‐Tropic Properties Unveiled in 3D Models and In Vivo in Nude Rats

**Figure 3:**
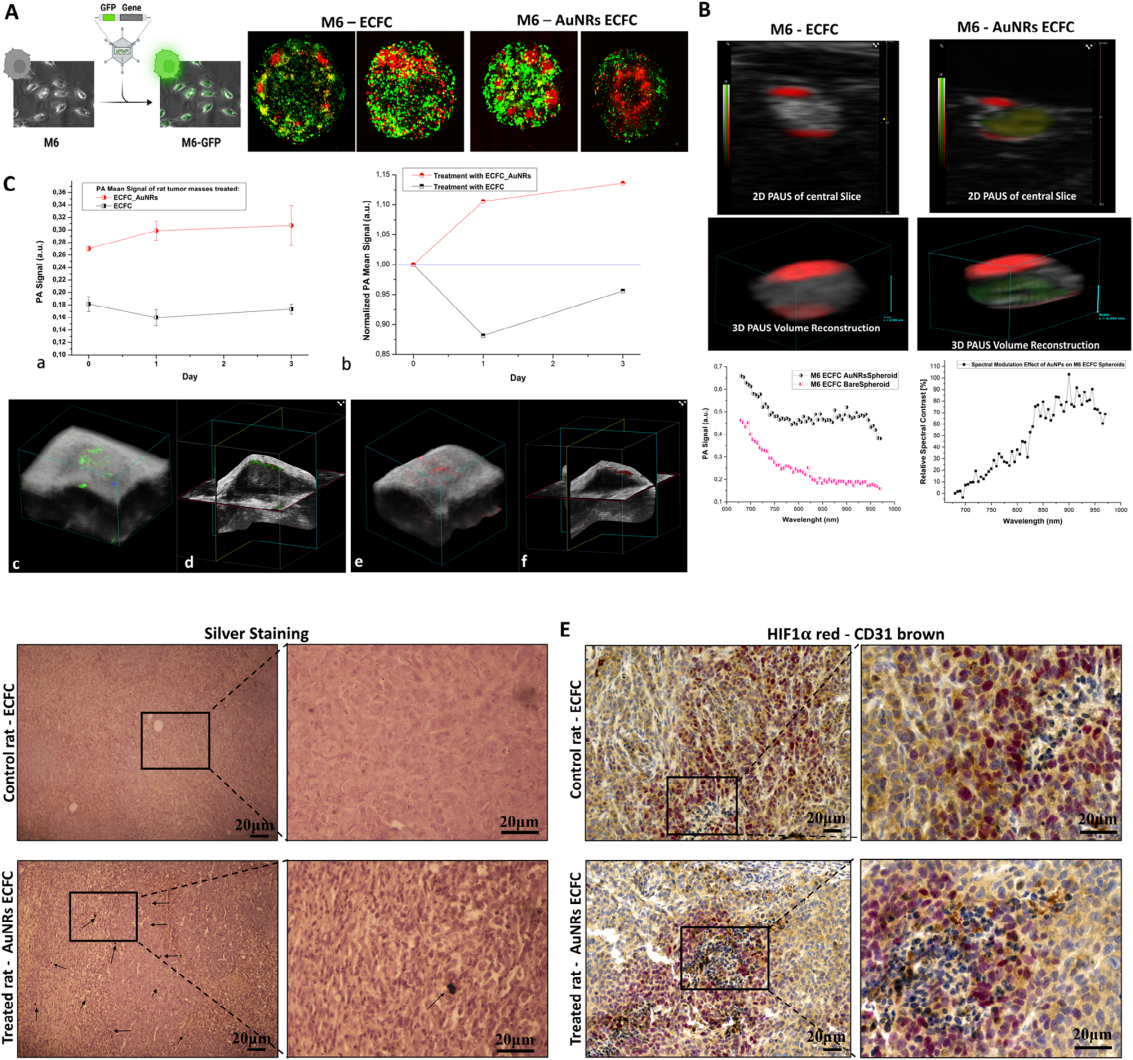
Integrated Confocal, Photoacoustic, and Histological Analysis of Tumor Spheroids with GFP‐Transfected Melanoma Cells and ECFCs and tumor mass. A) Confocal microscopy images of tumor spheroids composed of M6 GFP melanoma cells (green) and ECFCs, which are either naïve or enriched with AuNRs. ECFCs are stained with PKH26 (red). Scale bars represent 50 µm as indicated. The inset on the left shows optical images of an M6 cell monolayer before and after GFP transfection, confirming the successful incorporation of the green fluorescent protein. B) In the first raw, the photoacoustic acquisitions of a central slice of M6‐ECFC (dx) and M6‐AuNRs ECFC (sx), in the second raw the 3D PAUS volume reconstruction of the all tumor spheroids structure. The grayscale represent the US signal, the color scales (yellow, green, and red) represent the PA signal provided at different laser stimulation wavelength (970924680). On the bottom left specific photoacoustic signal intensity related to tumor spheroids with ECFCs enriched with AuNRs and those with naïve ECFCs, on the bottom right spectral photoacoustic comparison between a spheroid loaded with gold nanoparticles (AuNPs) and a bare control spheroid, where the photoacoustic signals were normalized prior to computing the relative difference according to the formula reported in the plot the normalized photoacoustic signals. This highlights wavelength regions where AuNPs significantly modulate the photoacoustic response C) panel a: PA signal intensity comparison of the mean values of the AuNR‐ECFC treated and non‐treated tumor masses during time, panel b: Normalized PA signal intensity from day 0 to day 3 of the treated and non‐treated tumor masses, panel c: 3D PAUS unmixed volume reconstruction of a referring treated tumor (gray scale for US, green color for the AuNR‐ECFCs, blue color for deoxygenated hemoglobin, red color for oxygenated hemoglobin), and panel d: the related 3D cross view plane visualization, panel e: 3D PAUS unmixed volume reconstruction of a referring treated tumor with naïve ECFCs (gray scale for US, blue color for deoxygenated hemoglobin, red color for oxygenated hemoglobin), and panel f: the related 3D cross view plane visualization. D) Representative images of histological assessments of rat tumor tissues one week after intravenous injection of naïve ECFCs or AuNR‐ECFCs, stained with silver to visualize gold nanorods (AuNRs), which appear as black precipitates. Black arrows indicate the presence of silver‐enhanced gold particles, primarily localized within the tumor microenvironment. E) Representative images of histological sections of the tumor masses stained with human CD31 (brown) and HIF‐1 alpha (red). The sections were counterstained with hematoxylin to visualize the nuclei. At the bottom enlarged views of the upper histological images, providing detailed visualization of the staining patterns. Scale bars represent 20 µm as indicated.

Expanding on our earlier findings demonstrating the successful migration of ECFCs loaded with GNPs to tumor masses upon administration through the mice tail vein^[^ ^39^ ^]^ we embarked on a novel exploration. For the first time, we investigated the comparative kinetics of recruitment of ECFCs with and without AuNRs in 3D spheroids. In contrast to conventional 2D monolayer models, 3D spheroids offer a more faithful recapitulation of the spatial architecture observed in solid tumors. These spheroids not only mirror the cell‐cell interactions within the complex tumor microenvironment but also faithfully reproduce the establishment of crucial gradients, such as those related to oxygen, nutrient availability, and pH levels. Furthermore, 3D spheroids capture the nuanced metabolic dynamics, genetic expressions, and extracellular matrix (ECM) deposition profiles that are inherent to in vivo tumor settings. To date, the utilization of 3D tumor models has been pivotal in screening various candidate cancer therapies, encompassing chemotherapeutics and their combinations,^[^ ^12^, ^50^ ^]^ immunotherapies,^[^ ^51^ ^]^ and photothermal/photodynamic therapies.^[^ ^52^ ^]^ Despite these advancements the evaluation of advanced tumor tropic living therapies within 3D microtumors remains a relatively underexplored avenue.^[^ ^52^, ^53^, ^54^ ^]^ Melanoma M6 cells were transfected with a green fluorescent protein (GFP) before performing spheroids whereas ECFCs naive and enriched with 100 µM Au AuNRs were colored with PHK26, a red fluorescent vital dye before being plated on the tumor spheroid. Confocal microscopy images (**Figure** **3**A) showed a deeper internalization of the AuNR‐ECFCs inside the spheroids compared to naïve ones, that tend to stay at the model outskirt. PA imaging of the 3D models was performed to corroborate the observations made by confocal microscopy. Considering the previously reported timescale of ≈24 h for ECFC migration and homing in tumors in vivo,^[^ ^39^ ^]^ this innovative approach allows for the real‐time monitoring of the migration of theranostic nanoparticle‐loaded cells, providing a dynamic insight into the interplay between ECFCs and tumor spheroids. Notably, the model demonstrated a persistent and increasing migration of the AuNR‐laden cells toward the core of the spheroid over an extended period of up to 8 days post‐administration of AuNP treatment. This observation underscores the viability and effectiveness of the nano‐in‐cell approach, showcasing the prolonged ability of these cells to infiltrate tumor spheroids. As shown in Figure 3B, the PA signal of gold, represented by the green area inside the spheroid, falls exactly in the AuNR spectrum of optical absorption, thus confirming that AuNR‐enriched ECFCs were able to reach the core of the sphere. Additionally, we assessed the presence of these cargo cells in nude rats, aiming not only to confirm their recruitment but also to map their localization within the tumor area. Following the subcutaneous injection of melanoma M6 cells into the flanks of nude rats, we allowed the tumor masses to form. Subsequently, ECFCs were injected into the caudal vein. Control rats received injections of naive ECFCs, while treated rats were administered with ECFCs enriched with AuNRs. The analysis of the entire tumor region clearly showed an increase in the PA signal over different time points in the treated rats compared to the controls (Figure 3C). This confirms the trend of recruitment of the ECFCs loaded with AuNRs into the tumor mass after a single injection. The temporal trend of cumulative PA signals obtained from tumor masses of mice treated with AuNR‐ECFCs showed mean values increasing from 0.27 ± 0.004 (a.u.) on day 0 to 0.31 ± 0.032 (a.u.) on day 3, while in the control tumor masses, the PA signal decreased from 0.18 ± 0.012 (a.u.) to 0.17 ± 0.008 (a.u.) (Panel a). This behavior has been highlighted by normalizing the dataset value with the PA value on day 0 (Panel b). The statistical analysis using one‐way ANOVA of the dataset of treated and untreated tumor masses showed a significant difference in mean values for each time point. (*p* < 0.05). Figure 3C shows the PA 3D unmixed volume reconstructions of two referring samples respectively a AuNR‐ECFC treated tumor mass (Panels c,d) and a control (Panels e,f), in order to visualize the volumetric distribution of the different components detected by the spectral unmixing tool. In our observations, as illustrated in Figure 3C, the cumulative photoacoustic (PA) signal values in tumor regions treated with gold nanorod‐embedded endothelial colony‐forming cells (AuNR‐ECFCs) were significantly higher on the day of injection compared to those treated with ECFCs alone. However, it is crucial to consider that the time required for ECFCs to localize, identify, and infiltrate the tumor masses may influence the interpretation of these results. Therefore, the PA signal recorded within the tumor volume on day 0 should be approached with caution, as it may not accurately reflect the specific accumulation of AuNRs within the tumor tissue. Rather, it is likely indicative of the overall presence of AuNRs within the tumor's vascular compartment. Notably, the intravenous bolus volume administered is 500 µL, representing ≈5% of the rat's total blood volume. Consistently, PA signal at day 0 exhibits a narrow range of values for all AuNR‐ECFC treated rats, as confirmed by the ANOVA 1way statistical analysis (*p* < 0.05). Subsequently, the presence of ECFCs was further validated through immunohistochemical analysis conducted one week post‐ECFC injection. Figure 3D displays hematoxylin and eosin staining, emphasizing the accumulation of AuNRs in the tumor masses of treated rats (indicated by black arrows). In contrast, microscopic examination of H&E‐stained liver, kidney, lung and spleen sections revealed preserved tissue architecture, with no evidence of necrosis, inflammation, or structural abnormalities, supporting the absence of treatment‐related toxicity in nude rats (Figure S4, Supporting Information). More significantly, our in vivo experiments enabled a deeper exploration. We were not only able to confirm the presence of ECFCs but also to precisely localize them in specific areas of the tumors. Hypoxia is one of the main features of solid tumors and was shown to correlate with poor prognosis in cancer patients because of its role in the phenomenon of therapy resistance. With the aim of assessing whether ECFCs were capable of reaching hypoxic areas, we conducted an immunohistochemical analysis for HIF‐1 alpha, a marker of hypoxia highlighted with RED staining, and CD31, a marker of endothelial cells, highlighted with DAB staining. As illustrated in Figure 3E, both naive and AuNR‐enriched ECFCs demonstrated a remarkable ability to reach the hypoxic areas within the tumor mass. While a few reports have demonstrated that hypoxia‐conditioned Mesenchymal Stem Cells (MSCs) possess higher tumor tropism features,^[^ ^55^, ^56^ ^]^ several attempts based on membrane camouflage strategies have been made to address tumor hypoxia and enhance the therapeutic effects of anti‐cancer treatments.^[^ ^57^, ^58^, ^59^ ^]^ However, our study represents a significant advancement by reporting, for the first time, the ability of tumor‐tropic cells loaded with gold nanoparticles to specifically home hypoxic areas within tumors. This discovery provides a robust platform for enhancing the therapeutic efficacy of anti‐cancer treatments. By specifically targeting hypoxic regions within tumors, this strategy addresses a critical challenge in oncology. The innovative approach offers significant potential to improve treatment precision and efficacy by overcoming hypoxia‐associated barriers. These findings pave the way for the refinement and optimization of therapeutic strategies, representing a pivotal advancement toward more effective and targeted cancer therapies.

### 2.3 Exploring AuNR ECFCs’ antitumoral effects in 3D models and in vivo

**Figure 4:**
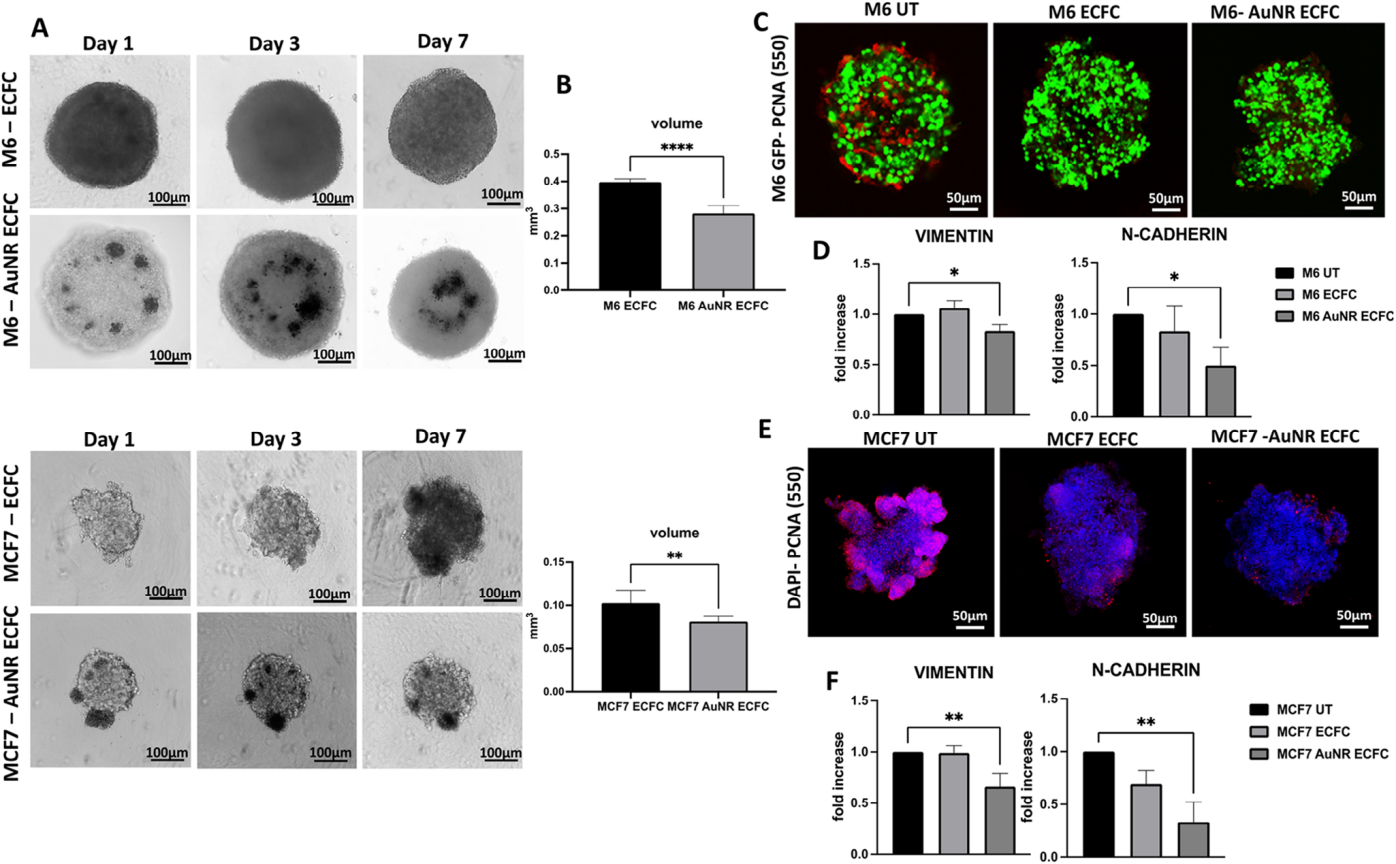
Multimodal Imaging and Gene Expression Analysis of Tumor Spheroids A) Optical images of tumor spheroids captured at the indicated time points, demonstrating the growth and morphological changes over time. Scale bars represent 100 µm as indicated. B) histogram illustrating the volume measurements of the tumor spheroids at day 7, providing quantitative data on spheroid growth. Confocal images of M6 GFP tumor spheroids C) and of MCF7 E) enriched with unstained naïve ECFC and AuNR‐ECFC with PCNA (Proliferating Cell Nuclear Antigen) stained in red, highlighting the proliferative cells within the spheroids. Scale bars represent 50 µm as indicated. D,F) Histograms showing the expression levels of vimentin and N‐cadherin genes in M6 and MCF‐7 cells, respectively, as measured by real‐time PCR. Significance was assessed by a one‐way ANOVA test followed by a Newman‐Keuls post‐test. Error bars indicate mean ± SD; asterisks (^*^ *p* < 0.05; ^**^ *p* < 0.001). All experiments were performed independently at least three times.

Building on our prior demonstration of the effect of endothelial colony‐forming cells on tumor growth and spread in a 2D model,^[^ ^39^ ^]^ this study extends our exploration. Here, we delve into the antitumoral effects of ECFCs enriched with AuNRs using advanced 3D tumor spheroids and in vivo model. To achieve the former objective, we established tumor spheroids representing two out of the four cell lines, specifically one for melanoma (M6) and one for breast cancer (MCF7). This selection was based on our prior demonstration of their overlapping thermal and photoacoustic behavior, ensuring a focused and representative model for our research. Tumor spheroids treated with both naive and AuNR‐enriched ECFCs underwent daily monitoring and photographic documentation (**Figure** **4**A) prior to volume measurements (Figure 4B). Surprisingly, both melanoma M6 and breast cancer MCF7 spheroids exhibited a significantly reduced volume when treated with AuNR‐ECFCs compared to those treated with naive ECFCs, as illustrated in Figure 4B. The assessment of tumor growth was then evaluated through both immunofluorescence analysis and real‐time PCR targeting PCNA (proliferating cell nuclear antigen). PCNA, a nuclear nonhistone protein crucial for DNA synthesis, serves as an accessory protein for DNA polymerase alpha and is elevated during the G1/S phase of the cell cycle. As depicted in Figure 4C,E, both M6 and MCF7 cells treated with AuNR‐ECFCs exhibited a reduced expression of PCNA. In addition, we also investigated whether ECFCs loaded with AuNRs could modulate the expression of markers involved in tumor progression and metastasis, specifically epithelial‐mesenchymal transition (EMT) related markers. Among the diverse EMT markers, including N‐cadherin, E‐cadherin, vimentin, Slug, Snail, Twist, and Zeb, our focus centered on investigating the expression levels of the two primary features, namely vimentin and N‐cadherin. As illustrated in panels 4D and 4F, a noticeable reduction in the expression of vimentin and N‐cadherin was observed in spheroids treated with ECFCs loaded with AuNRs for both cell lines.

**Figure 5:**
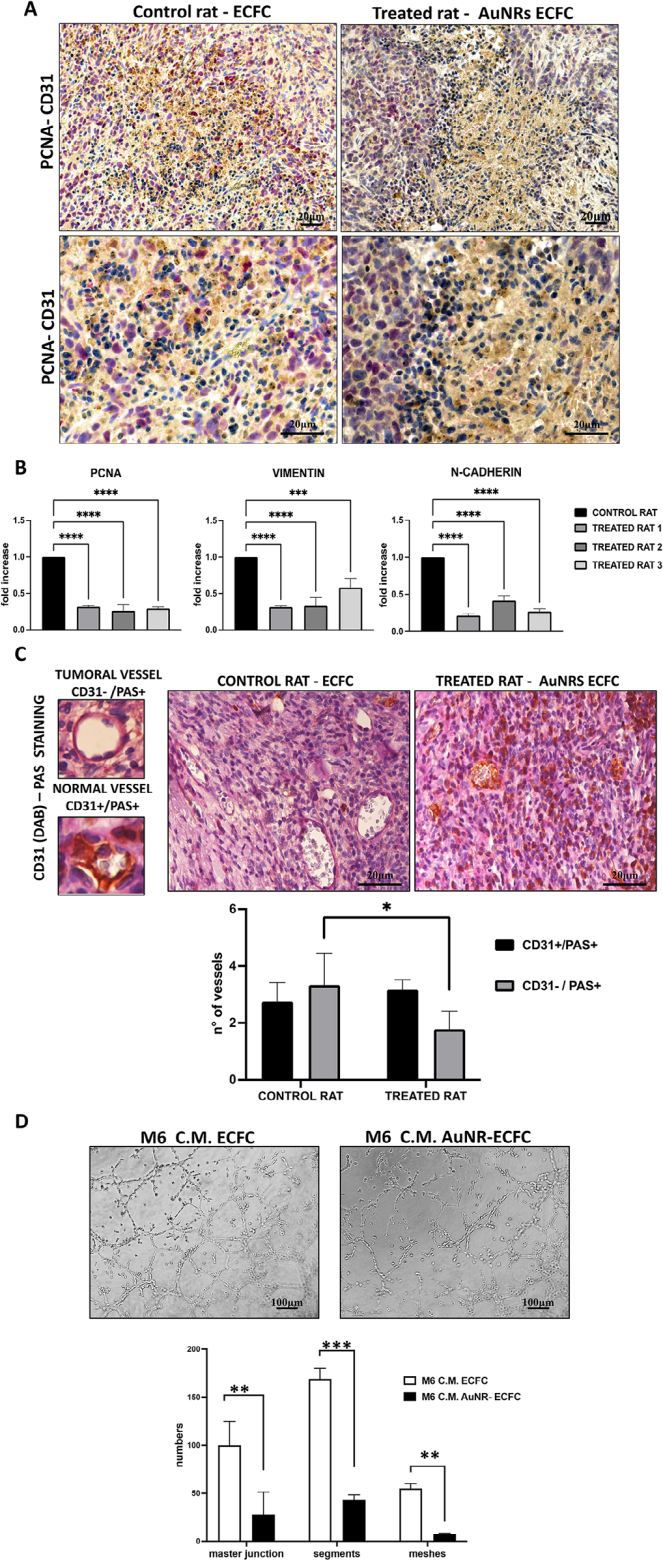
Histological and Gene Expression Analysis of Tumor Mass. (A) Representative images of histological sections of the tumor mass stained with human CD31 (brown) to indicate endothelial cells and PCNA (red) to highlight proliferating cells. Sections were counterstained with hematoxylin to visualize cell nuclei (blue). Scale bars represent 20 µm as indicated. (B) Histogram displaying the expression levels of Vimentin, N‐cadherin, and PCNA genes as measured by real‐time PCR. This provides insights into the mesenchymal and proliferative markers in tumor mass. Significance was assessed by a one‐way ANOVA test followed by a Newman‐Keuls post‐test. Error bars indicate mean ± SD; asterisks (*p < 0.05). (C) Representative images of histological sections of the tumor mass stained with human CD31 (brown) and PAS (Periodic Acid‐Schiff, pink) to indicate glycogen and other carbohydrate‐rich structures. The accompanying histogram quantitatively represents the number of vessels positive for CD31 alone and those positive for both CD31 and PAS, highlighting the vascular characteristics of the tumor. Scale bars represent 20 µm as indicated.

The evaluation of the antitumoral effects of AuNR‐ECFCs was assessed also in the tumor masses with a double immunohistochemical analysis for CD31, highlighted with DAB staining (brown), and PCNA, highlighted with RED staining. Again, it is possible to appreciate a noticeable decrease in the expression of PCNA‐positive nuclei when rats were treated with AuNR‐ECFCs, as displayed in **Figure** **5**A. The expression of PCNA in the tumor masses was also measured by real‐time PCR: rats treated with AuNR‐ECFCs showed a significant decrease in the gene expression of PCNA compared to control rats (Figure 5B), thus confirming the anti‐proliferative effects already shown in the immunohistochemical analysis. As shown in Figure 5B, it was also evaluated the gene expression of two main characters of the EMT transition: N‐cadherin and Vimentin resulted in down‐regulated when rats were treated with AuNR‐ECFCs. A critical factor contributing to tumor spread and growth involves the capability of tumors to form new vessels. It is well‐established that various malignant tumors, including metastatic melanoma, exhibit alternative mechanisms for blood supply within tumor tissues, alongside traditional angiogenesis. One such mechanism is known as “vasculogenic” or “vascular mimicry” (VM), and its presence has been correlated with an increased risk of metastasis and poor clinical outcomes. While traditional tumor angiogenesis relies on the growth of new blood vessels from pre‐existing host endothelium, VM involves the formation of a vascular‐like network directly by tumor cells. This distinction makes VM less responsive to traditional anti‐angiogenic therapies, contributing to the limitations observed in such treatments. We conducted in vivo investigations to ascertain whether the presence of ECFCs loaded with AuNRs could indeed modulate VM. The VM structures in the tumor areas are characterized by being rich in laminin and positive for Periodic Acid‐Schiff (PAS) staining while being negative for the endothelial marker CD31 staining. To scrutinize VM structures, we employed CD31‐PAS double staining, as illustrated in Figure 4C. Conventional blood vessels exhibited positive CD31 labeling (DAB staining) in their endothelial cells and a PAS‐positive reaction on the walls surrounding these cells. In contrast, VM vessels displayed a CD31‐negative label and a PAS‐positive reaction. Figure 5C and Figure S5 (Supporting Information) representing the tumor areas co‐stained with PAS and CD31, show that the AuNR‐loaded ECFC groups have significantly lower PAS‐positive areas than the control group indicating that the AuNR‐ECFCs decrease the formation of VM in the melanoma cancer xenografts. We confirmed the ability of ECFCs to inhibit the formation of tumor vessel structures in vitro using a capillary morphogenesis assay. Melanoma cells are known to reproduce vascular mimicry, and our observations indicate that M6 cells treated with conditioned media from ECFCs show a reduced capacity to form these structures. This inhibitory effect is even more pronounced when the ECFCs are enriched with gold nanorods (AuNRs) (Figure 5D)

This dual effectiveness, demonstrated in influencing tumor growth and progression in vitro and in vivo and specifically targeting VM, signifies a promising avenue for therapeutic applications. By concentrating on the modulation of VM, we can overcome the limitations associated with conventional anti‐angiogenic approaches.

(D) Capillary Representative microphotographs (x20) of capillary‐like structures at 24h formed by M6 untreated or treated with the conditioned medium from naïve ECFC or the conditioned medium from AuNR‐ECFCs. Scale bars represent 100 µm as indicated. The capillary network was quantified by Angiogenesis Analyzer Image J tool. Histograms represent the mean number of master junctions, segments, meshes, respectively. Data shown are representative of measures obtained from at least nine fields. Significance was assessed by a one‐way ANOVA test followed by a Newman‐Keuls post‐test. Error bars indicate mean ± SD; asterisks (^*^ *p* < 0.05, ^**^ *p* < 0.001, ^***^ *p* < 0.0001).

### 2.4 Enhanced Ferroptosis Induction in Melanoma Cells through Synergistic Hyperthermia and Radiotherapy

The capacity of ECFCs loaded with AuNRs to engraft within tumor masses, reaching hypoxic areas opens up the possibility of combining hyperthermia (HT) with radiotherapy (RT). Indeed, as already known in the literature, the addition of HT to ionizing radiation results in a synergic effect that influences several molecular parameters involved in sensitizing tumor cells to radiation, thus enhancing the potential of targeted radiotherapy. This becomes particularly relevant considering the radioresistance commonly associated with hypoxic tumor areas.

**Figure 6:**
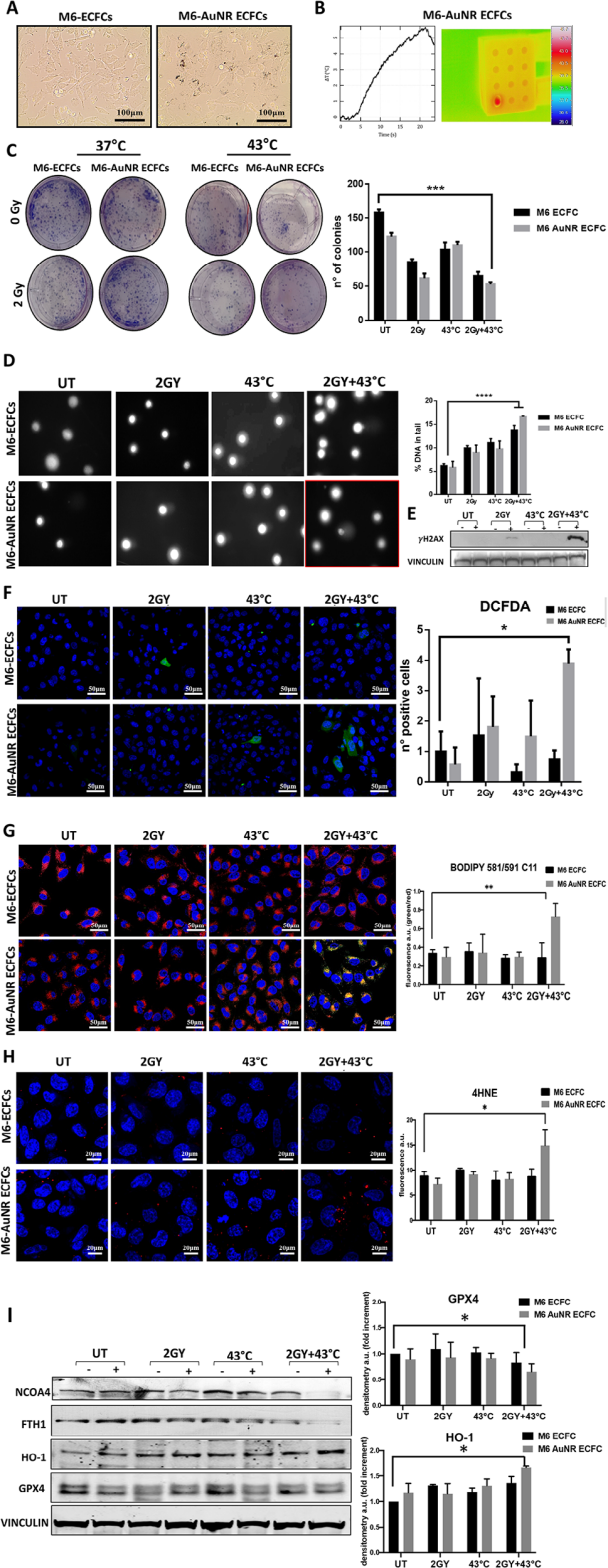
Multimodal analysis of melanoma cells M6 co‐cultures with naïve or AuNR‐treated ECFCs under hyperthermia, radiotherapy, or combined treatment. A) Representative optical microscope images of M6 melanoma cells co‐cultured with naïve ECFCs or AuNR‐treated ECFCs at a ratio of 5:1.‐. Scale bars represent 100 µm as indicated. B) Example of thermographs recorded after exposing M6/ECFC‐AuNR co‐cultures to 808 nm laser light with a power density of 1 W/cm^2^. On the right the thermographic image of the temperature detected on the surface of the irradiated well when the co‐culture reached 43 °C. C) Optical images of the colonies formed after 10 days by the M6/ECFC co‐cultures subjected to the indicated treatments. Colonies were stained with May‐Grünwald and then counted. Statistical analysis was performed using one‐way Anova. Representative data from three independent experiments are shown (mean ± SD). Asterisks indicate significant differences (^*^ *p* < 0.0001) compared to untreated cells. D) Representative images of comet morphology of M6‐ECFCs after irradiation with a 2 Gy dose, hyperthermia, and combination treatment. The graphs on the right indicate the levels of DNA damage, expressed as the percentage of DNA in the tail. Significance was assessed by a one‐way ANOVA test followed by a Newman–Keuls post‐test. Error bars indicate mean ± SD; asterisks (^*^ *p* < 0.05) indicate significant differences E) Western blot analysis of γH2AX levels in M6‐ECFC co‐cultures. Vinculin was used as a loading control. F) Confocal images of co‐cultured cells subjected to the indicated treatments and stained with the DCFDA probe. Green fluorescence indicates superoxide production. G) Confocal microscopy images of co‐cultured cells labeled with BODIPY 581/591 for the detection of lipid peroxides. Scale bars represent 50 µm as indicated. Histograms reports the quantification of the BODIPY C11 (581/590) oxidation ratio of biological replicates. Error bars indicate mean ± SD; asterisks (^*^ *p* < 0.05) indicate significant differences. H) Determination of lipid peroxidation by 4‐HNE‐fluorescence analysis. IF analysis using anti‐4HNE‐ab (red) and DAPI (blue) showing increased HNE adducts in the co‐cultures that had undergone the combination treatment. Scale bars represent 20 µm as indicated.

Encouraged by the high photothermal efficiency of AuNR‐ECFCs, we evaluated the effects of the combo treatment (HT+RT) on the co‐culture of M6 cells and ECFC naïve and enriched with AuNRs. Co‐culture was set up by mixing M6 cells and ECFCs at a ratio 5:1, as shown in **Figure** **6**A. Then, the photothermal properties (PTP) of AuNR‐ECFCs were tested by measuring the temperature elevation under NIR laser irradiation (1.0 W cm^−2^) as illustrated in Figure 6B. The combination treatment was performed subjecting the cells to mild hyperthermia at 43 °C coupled with a low dose of radiation (2Gy). Notably, the radiotherapy dose of 2 Gy was established based on a dose‐response curve ranging from 2 to 8 Gy, determined through clonogenic assays conducted on all tumor cell lines and ECFCs (Figure S6, Supporting Information). To evaluate the sustained impact of the combined RT+HT treatment, a colony formation assay was employed, as depicted in Figure 6C. Notably, the results reveal a significant reduction in the number of colonies formed following the combination treatment compared to the colonies formed when the two treatments were administered separately. The cytotoxic effect of the combo treatment was also deepened by analyzing the DNA damage with the COMET assay. As depicted in Figure 6D, the combination treatment exhibits a markedly higher level of DNA damage compared to the control and the two single treatments. This underscores the enhanced efficacy of the combined approach in inducing DNA damage. Together with the COMET assay, the evaluation of the DNA damage was carried out by investigating the expression of the histone H2Ax, which is phosphorylated on serine 139 when DNA double‐strand breaks (DSBs) occur. The expression of the histone γH2AX was analyzed by Western Blot analysis. As shown in Figure 6E, the phosphorylation of H2AX was significantly higher when cells were subjected to the combo treatment. Given that HT and RT are both known to elevate the production of reactive oxygen species (ROS), we assessed ROS levels employing two complementary approaches. First, ROS production was quantified using the fluorogenic probe dichlorodihydrofluorescein diacetate (DCFDA). This probe is oxidized by ROS to generate dichlorofluorescein (DCF), a highly fluorescent green compound, as shown in Figure 6F. Additionally, lipid peroxidation was evaluated using the ratiometric fluorescent sensor BODIPY C11. Upon oxidation, BODIPY C11 shifts its fluorescence emission from red to green, providing a dynamic measure of oxidative damage (Figure 6G). Confocal microscopy images revealed a significant increase in ROS levels following HT+RT treatment. This elevated ROS production was associated with enhanced lipid peroxidation at the cell membrane, leading to the accumulation of 4‐hydroxynonenal (4‐HNE), a cytotoxic aldehyde (Figure 6H). The increased presence of 4‐HNE suggests substantial oxidative damage and the formation of protein adducts, potentially resulting in protein dysfunction and cell death. To further explore the implications of ROS‐induced damage, we focused on ferroptosis, a distinctive form of cell death characterized by iron‐dependent phospholipid peroxidation, a mechanism that remains not fully elucidated.^[^ ^44^ ^]^ In this complex process, glutathione peroxidases, particularly selenoprotein glutathione peroxidase 4 (GPX4), play a crucial role by converting glutathione to its oxidized form (GSSG) and simultaneously reducing lipid peroxides (L‐OOH) to their alcohol forms (L‐OH). Our Western blot analysis revealed a notable downregulation of GPX4 expression in melanoma cells subjected to HT+RT treatment (Figure 6I). This reduction in GPX4 levels indicates the induction of ferroptosis. We then examined key markers and regulators of the ferroptosis pathway, observing significant changes in ferritin and its regulator NCOA4. Specifically, we noted a marked decrease in the levels of ferritin heavy chain (FTH1) and increased degradation of NCOA4, which mediates ferritinophagy.^[^ ^60^, ^61^ ^]^ Ferritinophagy facilitates the degradation of ferritin, releasing free iron that exacerbates lipid peroxidation and supports ferroptosis. The decreased levels of FTH1 suggest increased ferritin degradation and subsequent iron release, further supporting the occurrence of ferroptosis. Additionally, we detected increased levels of heme oxygenase‐1 (HO‐1), an enzyme upregulated in response to oxidative stress and involved in the degradation of heme to biliverdin, free iron, and carbon monoxide. The elevated HO‐1 levels indicate an adaptive response to oxidative stress and iron release, aligning with the ferroptosis process. The results presented in this study, therefore, demonstrate that the integration of hyperthermia and ionizing radiation not only reduces colony formation and induces significant DNA damage but also appears to trigger ferroptosis, as evidenced by increased ROS production, lipid peroxidation, decreased GPX4 expression, increased NCOA4 degradation, reduced FTH1 levels, and elevated HO‐1 expression. These results underscore the potential of HT+RT, particularly with effector cells loaded with gold nanorods (AuNRs), inducing ferroptosis and offer a promising approach for targeted cancer therapy.

Histogram reports the quantification of red fluorescence (mean ± SD of three separate experiments). Significance was assessed by a one‐way ANOVA test followed by a Newman–Keuls post‐test. Error bars indicate mean ± SD; asterisks (*p < 0.05) indicate significant differences. (I) Western blot analysis of different ferroptosis markers. Vinculin was used as a loading control. Histograms show the ratio of densitometric values of glutathione peroxidase 4 (GPX4) and heme oxygenase 1 (HO‐1) to vinculin. Error bars indicate mean ± SD.

### 2.5 Inducing Cellular Damage and Inhibiting Autophagy through the Synergy of HT and RT in Breast Cancer Cells (MCF7)

**Figure 7:**
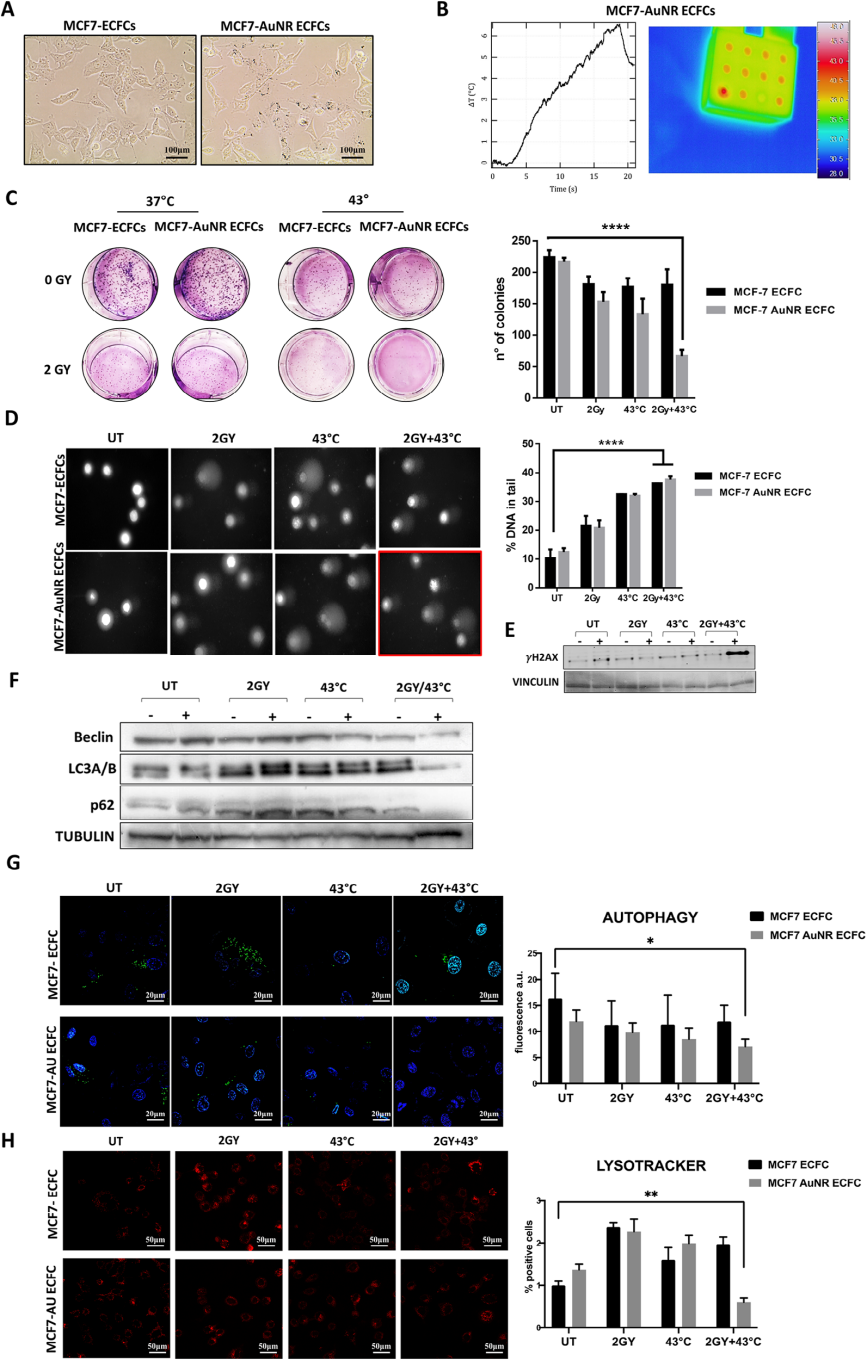
Multimodal analysis of breast cancer cells co‐coltures with naïve or AuNR‐treated ECFCs under hyperthermia, radiotherapy, or combined treatment. A) Representative optical microscope images of MCF7 cells co‐cultured with naïve ECFCs or AuNR‐treated ECFCs at a ratio of 5:1. Scale bars represent 100 µm as indicated B) Example of thermographs recorded after exposing MCF7/AuNR‐ECFC co‐cultures to 808 nm laser light with a power density of 1 W/cm^2^. On the right the thermographic images of the temperature detected on the surface of the irradiated well when the co‐culture reached 43 °C. C) Optical images of the colonies formed after 10 days by the MCF7/ECFC co‐cultures subjected to the indicated treatments. Colonies were stained with May‐Grünwald and then counted. Statistical analysis was performed using one‐way anova. Representative data from three independent experiments are shown (mean ± SD). Asterisks indicate significant differences (^*^ *p* < 0.0001) compared to untreated cells. D) Representative images of comet morphology of MCF7‐ECFCs after irradiation with a 2 Gy dose, hyperthermia, and combination treatment. The graphs on the right indicate the levels of DNA damage, expressed as the percentage of DNA in the tail. Significance was assessed by a one‐way ANOVA test followed by a Newman–Keuls post‐test. Error bars indicate mean ± SD; asterisks (^*^ *p* < 0.05) indicate significant differences E) Western blot analysis of γH2AX levels in M6‐ECFC co‐cultures. Vinculin was used as a loading control. G) Representative images of co‐cultures stained with CYTO‐ID Autophagy detection kit. Green fluorescence indicates selective positivity for the staining, which labels autophagosomes. Scale bars represent 20 µm as indicated. Histogram shows the quantification of the mean green fluorescence ± standard deviation of three independent experiments. H) After the treatments, co‐cultures were stained with BioTracker NIR633 Lysosome Dye (LysoTracker), a fluorescent stain for imaging lysosome localization and morphology in live cells. Scale bars represent 50 µm as indicated. Graphs represent the quantification of the number of red fluorescent lysosomes per cell. Significance was assessed by a one‐way ANOVA test followed by a Newman–Keuls post‐test. Error bars indicate mean ± SD; asterisks (^*^ *p* < 0.05, ^**^ *p* < 0.001) indicate significant differences.

Encouraged by the promising outcomes of the combined HT+RT treatment on melanoma cells, we extended our investigation to assess its effects on breast cancer cells. Specifically, we evaluated the treatment's impact on co‐cultures of MCF7 cells and ECFCs, both naïve and enriched with AuNRs. Co‐cultures were established by mixing MCF7 cells and ECFCs at a 5:1 ratio, as illustrated in **Figure** **7**A Then, the photothermal properties of AuNR‐ECFCs were tested by measuring the temperature elevation under NIR laser irradiation (1.0 W cm^−2^) as illustrated in Figure 7B. The combo treatment was performed with mild hyperthermia (43 °C) and with a low dose of radiation (2Gy) after evaluating the dose‐dependent long‐term effects of radiation, as shown in Figure S6 (Supporting Information). The long‐term effects of the combo treatment of radiotherapy and hyperthermia were evaluated with a colony formation assay, as depicted in Figure 7C, where the number of colonies formed after the combo treatment is significantly lower than the colonies formed with the two treatments carried out separately. The cytotoxic effect of the combo treatment was also deepened by analyzing the DNA damage with the COMET assay. Figure 7D highlights a notable increase in DNA damage resulting from the combination treatment compared to both the control and the individual treatments considered separately. This underscores the enhanced effectiveness of the combined approach in inducing DNA damage. Together with the COMET assay, the evaluation of the DNA damage was carried out by investigating the expression of the histone γH2Ax by Western Blot, as shown in Figure 7E. We also evaluated ferroptosis in this model, as shown in Figure S7 (Supporting Information), and contrary to expectations, the results demonstrated that the combination treatment of mild hyperthermia and low‐dose radiation was not effective in inducing cell death through the ferroptotic pathway. As already known in literature, there is a correlation between ferroptosis and autophagy.^[^ ^62^ ^]^ Indeed, autophagy plays an essential role in the induction of ferroptosis by regulating cellular iron homeostasis and ROS generation.^[^ ^63^ ^]^ Nevertheless, autophagy helps cells maintain homeostasis under stressful conditions by degrading and recycling unnecessary or dysfunctional organelles and proteins in a double‐membraned vacuole known as an autophagosome. Recently, beyond the hallmarks of cancer originally proposed by Weinberg et al.,^[^ ^64^ ^]^ new fundamental hallmarks have been identified, including the ability to tolerate metabolic, oxidative, DNA damage, mitotic, and proteotoxic stresses.^[^ ^65^ ^]^ Given that autophagy can help tumor cells endure these various stresses, evaluating autophagy's role in response to specific treatments will likely enhance therapeutic effectiveness. Many agents under development in clinical trials have been found to modulate autophagy, such as histone deacetylase inhibitors, anti‐angiogenic agents, mTOR inhibitors, BH3 domain mimetics, and glycolytic inhibitors.^[^ ^66^, ^67^ ^]^

In our study, the combination of hyperthermia and radiotherapy resulted in a significant inhibition of autophagy in MCF‐7 cells. This inhibition was indicated by a substantial reduction in the levels of LC3 I‐II and Beclin‐1, accompanied by the complete disappearance of p62 (Figure 7F). Autophagy, a dynamic process involving the formation and maturation of structures such as phagophores, autophagosomes, and autolysosomes, was further assessed using CYTO‐ID Autophagy detection kit,^[^ ^68^ ^]^ which revealed a marked decrease in the number of green punctate autophagosomes (Figure 7G). Recent genetic studies suggest that while LC3B/Atg8 may not be essential for autophagosome biogenesis, it is crucial for the fusion of autophagosomes with lysosomes.^[^ ^69^ ^]^ The observed decrease in autophagosomes correlated with reduced LysoTracker staining intensity, which reflects lysosomal biogenesis and/or increased lysosomal activity.^[^ ^70^ ^]^ As depicted in Figure 7H, MCF‐7 cells treated with the HT and RT combination showed diminished LysoTracker staining, consistent with the Western blot and Enzo staining results. This reduction in lysosomal activity underscores the effective inhibition of autophagy by the combined treatment. Such inhibition of autophagy may potentially counteract radioresistance and chemoresistance and re‐sensitize cancer cells to therapies.

## 3 Discussion

Radiotherapy (RT) has long been recognized as a crucial therapeutic modality in the management of metastatic cancers,^[^ ^9^ ^]^ due to its ability to directly induce DNA damage and generate reactive oxygen species (ROS) to hinder tumor proliferation.^[^ ^71^, ^72^, ^73^ ^]^ However, its efficacy is often compromised by challenges such as tumor hypoxia and inherent resistance mechanisms. The integration of hyperthermia (HT) with RT has emerged as a promising approach to mitigate these limitations, enhancing radiosensitivity and improving therapeutic outcomes.^[^ ^74^, ^75^ ^]^ Our study introduces a novel strategy utilizing endothelial colony‐forming cells (ECFCs) as a targeted delivery system for gold nanorods (AuNRs), thereby enhancing RT through hyperthermia and overcoming hypoxia‐driven resistance.

Based on our in vitro and in vivo investigations, we demonstate that ECFCs loaded with AuNRs precisely localize to hypoxic tumor regions, significantly inhibiting tumor growth and vascular mimicry. This targeted approach ensures a potent therapeutic impact while minimizing systemic toxicity. The combined RT and HT strategy activates distinct cancer‐specific mechanisms: in melanoma cells, it induces ferroptosis, a form of programmed cell death dependent on iron and ROS accumulation; in breast cancer cells, it inhibits autophagy, a process implicated in therapy resistance. These findings underscore the importance of tailored interventions based on tumor biology and highlight the translational potential of ECFC‐mediated AuNR delivery.

Our study builds upon previous research phases exploring RT + HT combination therapy. The initial research phase primarily focused on the identification of synergistic effects between RT and HT without elucidating underlying mechanisms.^[^ ^27^ ^]^ Subsequent investigations examined hypoxia‐induced DNA damage, cell cycle arrest, and apoptosis regulation.^[^ ^76^, ^77^, ^78^ ^]^ In the most recent research phase, studies have sought to optimize RT + HT conditions and explore immune modulation as a potential therapeutic avenue.^[^ ^79^, ^80^ ^]^ Our findings contribute to this evolving field by providing, for the first time, a detailed mechanistic insight into how RT + HT modulates different cell death pathways across distinct cancer types. Notably, our results demonstrate significant inhibition of autophagy in MCF‐7 cells upon RT + HT treatment, as indicated by reduced LC3 I‐II and Beclin‐1 levels and the complete absence of p62. The observed reduction in autophagosomes, as evidenced by CYTO‐ID Autophagy detection assays, aligns with decreased lysosomal activity, confirmed by diminished LysoTracker staining. This suggests that autophagy inhibition may re‐sensitize resistant cancer cells to therapy, providing a novel approach to overcoming treatment resistance.

Additionally, our study extends prior knowledge by integrating 3D tumor models and in vivo analyses using nude rats, ensuring that our findings have robust translational relevance. The ability of ECFCs to efficiently deliver AuNRs to hypoxic regions and enhance RT + HT effects highlights the potential of cell‐mediated nanoparticle therapies in precision oncology. Our approach surpasses conventional nanoparticle‐based strategies by ensuring enhanced tumor penetration and sustained therapeutic impact.

Given the growing interest in immunotherapy, future investigations should explore the immune‐modulatory effects of RT + HT in combination with AuNR delivery. Understanding how this strategy influences tumor immunogenicity and immune cell infiltration will be critical in designing combinatory treatment regimens. Moreover, optimizing the temporal and spatial dynamics of ECFC‐mediated AuNR delivery may further refine treatment efficacy and minimize potential off‐target effects.

**Figure 8:**
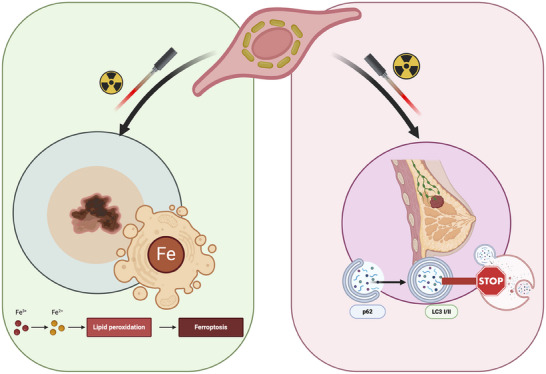
Schematic representation of the mechanistic interplay of hyperthermia and radiotherapy in two different cancer types, created in Biorender.

In conclusion, our study provides compelling evidence that ECFC‐mediated AuNR delivery enhances RT through hyperthermia, offering a targeted and potent therapeutic strategy for metastatic cancers. By elucidating the distinct cellular responses in melanoma and breast cancer (**Figure** **8**), we pave the way for personalized interventions that address tumor heterogeneity and therapeutic resistance. This novel approach holds significant promise in advancing RT + HT combination therapy and expanding its applicability in clinical oncology.

## 4 Experimental Section

### Cell Lines

ECFCs, a subpopulation of EPCs, were isolated from >50 mL human UCB of healthy newborns, as described in ref. (X) after maternal informed consent and in compliance with Italian legislation and analyzed for the expression of surface antigens (CD45, CD34, CD31, CD105, ULEX, vWF, KDR, and uPAR) by flow cytometry. ECFCs were cultured in EGM2 medium (Lonza, Euroclone) supplemented with 10% (v/v) fetal bovine serum (FBS, Euroclone). A375‐M6 melanoma cells (M6) were isolated in the laboratory from lung metastasis of SCID bg/bg mice i.v. injected with A375 melanoma cells. A375 cells obtained from American Type Culture Collection (Manassas, VA), and M6 cells were independently validated by STR profiling at the DNA diagnostic center BMRGenomics (Padova, Italy). Cells were amplified, stocked, and once thawed were kept in culture for a maximum of 4 months. The melanoma A375 cell line, the human breast cancer cell line MDA‐MB 231 and MCF‐7 were obtained from American Type Culture Collection (Manassas, VA) and grown in Dulbecco's modified Eagle's medium (DMEM) with 10% FBS (Euroclone) and 1% l‐glutamine in a humidified 5% CO_2_ incubator maintained at 37 °C (Thermo Scientific, Waltham, MA).

### Gold Nanorods (AuNRs) Preparation

Gold nanorods (AuNRs) with a longitudinal mode of plasmonic oscillations in the first biological window were synthesized and stabilized with cetrimonium bromide (CTAB), according to the method reported by Ye et al for a band peaking ≈810 nm.^[^ ^81^ ^]^ CTAB is a crucial surfactant in gold nanorod synthesis, employed either alone or in combination with other dispersants^[^ ^82^ ^]^ as it uniquely fulfills both the roles of shape‐directing agent and colloidal stabilizer.

In order to improve their biocompatibility and to promote their intracellular uptake, AuNRs were gradually coated with low‐molecular weight chitosan, through a layer‐by‐layer approach with an intermediate shell of poly (sodium 4‐styrenesulfonate) (Na+‐PSS, 70 kDa). While alternative anionic polyelectrolytes, such as synthetic polymers like polyacrylic acid and polyvinyl sulfate^[^ ^83^ ^]^ or natural polysaccharides like alginate, may be considered, the use of PSS for gold nanorod modification benefits from a robust established background that we have successfully optimized in our previous work.^[^ ^84^ ^]^ Here the replacement of CTAB with PSS was achieved by following the procedure reported by Mehtala et al ^[^ ^85^ ^]^ with modifications. In particular, the as‐synthesized CTAB‐stabilized AuNRs (1.6 mM Au) were transferred to an aqueous solution of 0.15% Na+‐PSS also containing 0.5 mM CTAB for 24 h at RT under a gentle stirring, which proved to improve the reproducibility and stability of the colloidal system. Particles were then purified by three cycles of centrifugation and resuspension with 0.15% Na+‐PSS without additional CTAB, and then transferred to a solution of 0.15% chitosan with 1.5% citric acid for a further 24 h. Finally, chitosan‐coated AuNRs were washed with ultrapure water, sterilized by a standard autoclave procedure, and finally stored at 4 °C at a final concentration of 4 mM Au before implementation in all subsequent biological tests. Particles were characterized by dynamic light scattering (DLS) (NANO‐ZS90 Zetasizer, Malvern Instruments, UK) and UV‐visible/NIR spectrophotometry (V‐770 UV‐Visible/NIR Spectrophotometer, Jasco, Japan).

### Nanoparticle Tracking Analysis

A nanoparticle tracking analysis (NTA) was performed, as previously described,^[^ ^86^ ^]^ with a NanoSight NS300 (Malvern Panalytical, Westborough, MA, USA) apparatus equipped with a 488‐nm excitation laser and an automated syringe sampler. The NTA technology calculates the size distribution, the total concentration of the sample, the relative scattering intensity, and the fluorescence of each particle through the Stokes–Einstein equation based on the relationship between the Brownian motion and hydrodynamic diameter. Nanoparticles were diluted in PBS to a final volume of 1 mL. Optimal concentrations were found by pre‐testing the ideal particle per frame value. After ideal concentration setting, all samples were diluted at 1:50 in PBS and then loaded into syringes. For each measurement, five cycles were performed by capturing 60 frames per position under the following conditions: cell temperature: 25 °C; Syringe speed: 30 µL s^−1^. The total number tracks in NTA measurements was greater than the proposed minimum of 1000 in order to minimize data skewing based on single large particles. CSV files generated by NTA by software v3.2 were used for a computational analysis.

### Inductively Coupled Plasma Atomic Emission Spectroscopy (ICP‐AES)

For the uptake's kinetic study, ECFCs, A375, M6, MCF‐7 and MDA (3.0 × 10^5^) were seeded on 10 cm dishes and allowed to attach overnight. On the next day, cells were incubated with culture medium containing AuNRs at the concentration 100 × 10^−6^ M Au for the indicated time points. Cells undergoing the double administration (o/n + 3h) were washed with PBS (Invitrogen) and treated again with culture medium containing AuNRs (100 × 10^−6^ M Au) for 3 h. Cells were then washed two times with PBS (Invitrogen), detached with a trypsin treatment, and counted using a hemocytometer. Cell pellets were collected by centrifugation, lyophilized, and placed in centrifuge tubes (one pellet per tube). Then, 400 µL of aqua regia was added to each tube to completely dissolve the cells and their gold content. The amount of Au was measured by Elan DRC II ICP‐MS (Perkin Elmer, Waltham, MA).

### TEM Analysis of AuNRs Enriched Cell Lines and ECFCs

ECFCs, A375, M6, MCF‐7 and MDA were seeded in 6‐well plates at a density of 1.5 × 10^5^ cells per well and allowed to reach 70% confluence. Next, cells were incubated with culture medium (2 mL per well) containing suspensions of AuNRs at a concentration of 100 × 10^−6^ M Au for the indicated time points, then collected by trypsin treatment, and centrifuged at 1000 rpm for 5 min in a 1.5 mL Eppendorf tube. The cell pellet was then fixed in isotonic 4% glutaraldehyde and 1% OsO4, dehydrated, and embedded in Epon epoxy resin (Fluka, Buchs, Switzerland) for electron microscopy. Ultrathin sections were stained with aqueous uranyl acetate and alkaline bismuth subnitrate and viewed and photographed under a JEM 1010 transmission electron microscope (Jeol, Tokyo, Japan) equipped with a MegaView III high‐resolution digital camera and imaging software (Jeol).

### Photoacoustic Imaging In Vitro and In Vivo

PA experiment was performed using the multimodality imaging platform Vevo LAZR by FUJIFILM Visualsonics Inc. (Toronto). The PA properties and performances of ECFCs were evaluated in test‐object phantoms as previously reported.^[^ ^39^ ^]^ The test‐object for PA characterization consisted of a custom‐made phantom constructed from a polypropylene box containing coplanar polyethylene (PE) micrometric tubes. These tubes, with an internal diameter of 0.58 mm and an external diameter of 0.99 mm, were filled with 100 000 cells of ECFCs, MCF7, A375, MDA, or A375‐M6, each pre‐treated with 100 µM of AuNPs.Photoacoustic (PA) multispectral analysis was performed across the 680–970 nm wavelength range to capture their unique spectral fingerpri The PA multispectral analysis was conducted within the 680 to 970 nm range to identify their specific fingerprint. Regarding the in‐vivo experimentation, the treated tumors were injected by intravenous tail vein administration at day 0 and day 2 of 500 µL bolus of AuNR‐ECFCs (containing a total of 700.000 cells), while control masses were injected with a 500 µL bolus of ECFCs (850.000 cells). in vivo experiment was performed using the Vevo LAZR at three time points: day 0, day 1 and day 3. 3D distribution of the photoacoustic signal within the tumor region was acquired using the multiwavelength mode. In detail, tumor mass volumes were scanned at a step size of 203 µm, and for each slice the PA signal was retrieved at 6 different wavelengths (680, 730, 800, 924, 930 and 968 nm). The PA 3D acquisitions were processed to separate the mixed spectral signals of AuNR‐enriched ECFCs, endogenous responsive molecules (oxygenated hemoglobin, deoxygenated hemoglobin, and melanin), and noise into their individual contributions inside the samples. The characteristic spectra of each component against which this discrimination was performed were obtained as follows: for oxygenated and deoxygenated hemoglobin, default spectra provided by the software were used; for melanin, AuNRs and noise, spectra acquired from corresponding regions of interest were employed (skin for melanin, regions with air bubble presence for noise, and phantom test tubes for AuNRs). The PA signal values of each component were ultimately derived within a region of interest drown across the entire acquisition volume of the tumor mass, taking the PA average threshold, that averages the top 10% of values within the ROI. Due to the similarity exhibited by the spectra of AuNR‐ECFCs and the typical PA spectral trend of the endogenous molecule of oxygenated hemoglobin, to evaluate and validate the recruitment of AuNR‐ECFCs in the rat tumor masses, the cumulative PA signal of AuNR‐ECFCs and Oxygenated hemoglobin was compared with the controls during the timepoints at the day 0, day1, and day 3.

### Statistical Analysis for PA In‐Vivo AuNR‐ECFCs Accumulation

A Bayesian hierarchical model was employed to analyze longitudinal photoacoustic signal measurements from tumors treated with nanoparticles and untreated controls. Measurements were acquired at three time points: baseline (T0), and two follow‐up points (T1 and T2). The aim was to estimate both the intra‐group changes over time and the differential effect of treatment. Data consisted of measurements from 8 treated tumors and 2 untreated controls, each evaluated at T0, T1, and T2. For each group, the signal intensities at the three‐time points were modeled using Gaussian likelihoods with weakly informative Normal priors (μ ∼ Normal(0, 100), σ ∼ HalfNormal(50)). The model included a shared variance across time points to account for within‐group variability. The longitudinal change in signal for each group was defined as:

(1) Δt=μ_t−μ_0,fort=1,2

where μt is the estimated mean signal at time t, and μ0 is the baseline signal.

The treatment effect at each time point was defined as the difference in signal changes between the treated and control groups:

(2) $$\left[Effect\right]\_t=\left[\Delta t\right]\_treated-\left[\Delta t\right]\_control$$

The longitudinal change in signal was defined as the difference in group means between time 𝑡∈{1,2}, and baseline (T0). In treated tumours, the signal increased by an average of +28.3 units from T0 to T1 (95% credible interval: [0.2, 56.5]), and by +36.1 units from T0 to T2 (95% CI: [8.2, 64.2]), suggesting a consistent and progressive enhancement over time. In contrast, control tumours exhibited an average decrease of –20.6 units from T0 to T1 (95% CI: [–60.4, +24.7]) and a small, uncertain change of –7.7 units from T0 to T2 (95% CI: [–49.7, +38.3]). To quantify the treatment effect, we computed the difference in signal change between treated and control groups. The estimated treatment effect was +64.0 units at T1 (95% CI: [–5.8, +103.2]) and +43.7 units at T2 (95% CI: [–10.6, +100.8]). While the credible intervals slightly overlap zero, the posterior distributions are positively skewed and the bulk of probability mass lies above zero, indicating a strong tendency toward a beneficial treatment effect. Despite the limited sample size, particularly in the control group, the posterior estimates show a clear and credible intra‐group signal increase over time in the treated tumours. The controls, conversely, show no consistent change or even a decrease in signal. While the 95% credible intervals for treatment effects slightly overlap zero, the posterior distributions are highly right‐skewed, suggesting a strong tendency toward a positive nanoparticle accumulation at both time points. This supports the hypothesis that the optimized setup enables detection of nanoparticle‐mediated signal accumulation over time.

Posterior inference was performed using Markov Chain Monte Carlo (MCMC) sampling via PyMC (v5.9.0), with 2000 samples drawn after 1000 tuning steps. All convergence diagnostics were satisfactory (R̂ ≈ 1.0, effective sample sizes > 2000), confirming stable and reliable inference.


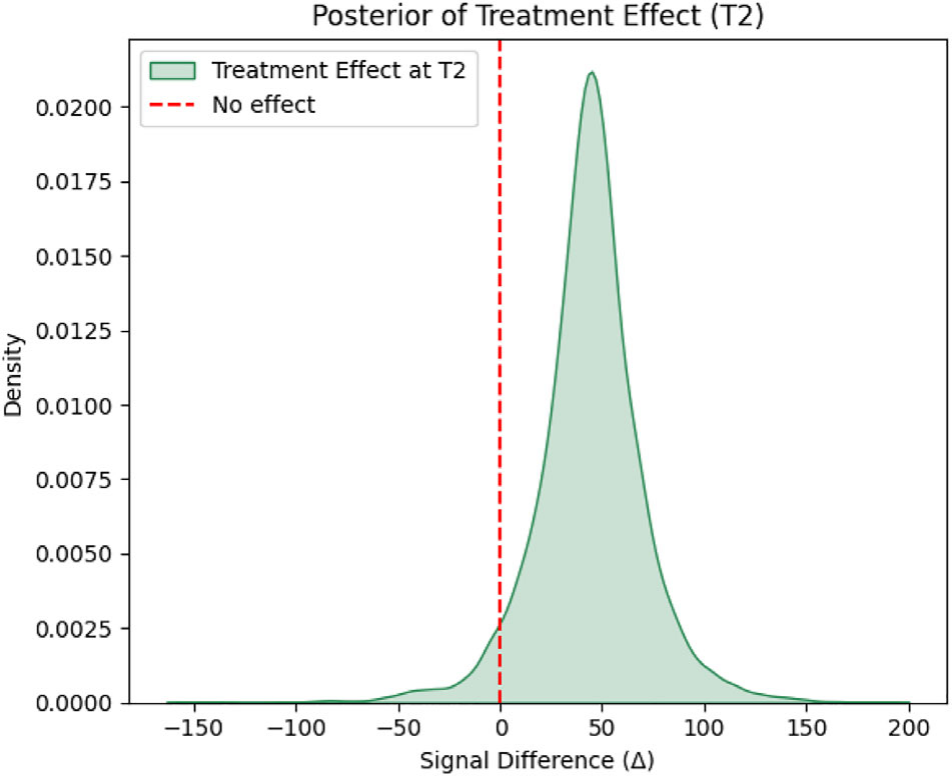


Stat1 1:Posterior distribution of the treatment effect at T2 The plot shows the posterior distribution of the difference in signal increase between the treated and control groups at time point T2 (Δ₂^treated − Δ₂^control). The distribution reflects the uncertainty in estimating the treatment effect, based on the observed data and model priors. The shaded area represents the kernel density estimate of the posterior samples. The red dashed line at zero denotes the null hypothesis of no treatment effect. The bulk of the distribution lies above zero, indicating a credible increase in signal attributable to treatment. The 95% highest density interval (HDI) excludes zero, supporting the presence of a treatment‐induced accumulation at T2.

### Photothermal Properties of AuNRs Enriched Cell Lines

ECFCs, A375, M6, MCF‐7, and MDA were seeded in 6‐well plates at a density of 1.5 × 10^5^ cells per well and allowed to reach 70% confluence. Cells were then treated with AuNRs at the concentration 100 × 10^−6^ M Au before being harvested by trypsin treatment and centrifuged at 1000 rpm for 5 min in a 1.5 mL Eppendorf tube. Cells were resuspended with 30 µL of medium before undergoing photothermal treatment. The cells were placed in a custom‐made multiwell plate connected to a chiller set at a temperature of 38 °C, in order to allow the cell suspensions to reach the physiological value of 37 °C before performing NIR laser irradiation. The samples were then irradiated with a DEKA mod Smarty A800 CW laser diode emitting at a wavelength of 808 nm with a power density of 1 W cm^−2^. Laser light was delivered through a multimode optical fiber and collimated onto a 5 mm diameter spot. The temperature increase was monitored during irradiation with a NEC Avio mod InfReC R300SR thermal imager (spectral range 8–14 µm, NETD 30 mK at 30 °C) equipped with a 10 cm focal length objective and a field of view of 22° × 17°. Images were processed with InfReC Analyzer NS9500 software.

### 3D Cultures

Melanoma M6 cells were transfected with GFP (VectorBuilder) using Lipofectamine 3000 Transfection Reagent (Thermofisher) according to manufacturer's instruction. M6‐GFP positive cells were selected thanks to puromycin resistance 2 µg mL^−1^ (Invivogen). Melanoma M6 and breast cancer MCF‐7 cells were detached by trypsin treatment and counted with a hemocytometer. 3 × 10^3^ cells were seeded in each well of an ultra‐low attachment (ULA) plate (Nuclon Sphera, Thermo Scientific) in 150 µl of complete medium and centrifuged at 800 rpm for 5 min. After 3 days, spheroids were co‐cultured with naïve and AuNR‐enriched ECFCs, stained with PKH26 (Sigma). 3D cultures were maintained in a humidified 5% CO_2_ incubator at 37 °C (Thermo Scientific, Waltham, MA). Spheroids were observed and imaged using a conventional optical microscope under white light exposure, Invitrogen EVOS XL Core Imaging System (Thermofisher). The volume of the spheroids was calculated with ImageJ software. The localization of ECFCs naïve and enriched with AuNRs inside the tumor sphere was analyzed with TCS SP8 microscope (Leica Microsystems) with LAS‐AF image acquisition software.

### 3D Confocal Microscopy Analysis

Spheroids were then fixed with 4% PFA overnight at +4 °C and stained with Cytovista 3D Cell Culture Clearing Kit (Thermofisher) according to manufacturer's protocol. Spheroids were analyzed for the expression of PCNA (1:100 Cell Signaling, #13 110) then stained with anti‐rabbit AlexaFluor550 (Invitrogen) for 1h at RT before staining Nuclei with DAPI (10 µg mL^−1^) (Sigma) for 15 min at 37 °C. Sample images were acquired using TCS SP8 microscope (Leica Microsystems) with LAS‐AF image acquisition software. Series of optical sections (XY: 1024 × 1024 pixels) were then taken through the depth of the spheroids with a thickness of 2000 µm at intervals of 10 µm (Z step). A single composite image was obtained by the superimposition of 20 optical sections for each sample.

### RNA Extraction and Quantitative PCR

Total RNA was prepared using Tri Reagent (Sigma‐Aldrich, Saint Louis, Missouri, USA), and reverse transcribed with cDNA synthesis kit (BioRad, Milano, Italy) according to the manufacturer's instructions. Selected genes were evaluated by real‐time PCR using SsoAdvanced Universal Green Mix (BioRad) with CFX96 Touch Real‐Time PCR Detection System (BioRad). For real‐time PCR, fold change was determined by the comparative Ct method using 18S‐rRNA as normalization gene. Amplification was performed with the default PCR setting: 40 cycles of 95 °C for 10 s and 60 °C for 30 s using SYBR Green‐based detection. Primer sequences (IDT, TemaRicerca) were as follows:

Human 18S‐rRNA: Forward CCAGTAAGTGCGGGTCATAAG and reverse GCCTCACATAA‐CCATCCAATC
Human N‐Cadherin: Forward CCTCCAGAGTTTACTGCCATGAC and reverse GTAGGATCTCCGCCACTGATTC
Human Vimentin forward AGGCAAAGCAGGAGTCCACTGA and reverse ATCTGGCGTTCCAGGGACTCAT

### In Vivo Experiments

All in vivo procedures were approved by the Animal Welfare Office of the Italian Ministry of Health (authorization number 541/2021‐PR) and were conducted in accordance with national legal requirements and Italian guidelines for the care and use of laboratory animals. Female athymic nude rats (RH‐Foxn1rnu), aged six to eight weeks, were purchased from Envigo. Melanoma tumor masses were induced by subcutaneously injecting 5 × 10^6^ M6 cells in the flanks of the nude rats. One week after tumors induction, rats were treated by injecting 1 × 10^6^ naive and AuNR‐enriched ECFCs in the caudal vein of rats. All these procedures were run under 2% isoflurane anesthesia. The animals were monitored daily and were sacrificed one week after the last ECFC injection. The histological analysis of tumor mass was performed as described below. Removed tumors and RES organs were fixed at 4 °C in 10% neutral buffered formalin (BioOptica) for histological analysis performed on paraffin‐embedded sections (5 µm). The sections of tumor tissue were stained using hematoxylin and eosin (BioOptica) and immunohistochemical analysis were performed with BOND‐MAX IHC (Leica) to evaluate the expression of CD31 (Thermofisher), HIF1a (Cell signaling), PCNA (Cell Signaling). The evaluation of vascular mimicry was performed with the PAS Schiff staining (BioOptica). All sections were examined using an optical microscope and photographs were acquired using Leica Scanner Aperio CS2 (Leica). To evaluate the presence of AuNRs inside the tumor masses, Silver Stain (Silver Enhancer Kit, Sigma) was performed according to manufacturer’ instructions.

### Western Blot Analysis

Harvested cells were resuspended in 20 × 10^−3^ M RIPA buffer (pH 7.4) (Merk Millipore, Vimodrone, MI, Italy) containing a cocktail of proteinase inhibitors (Calbiochem, Merck, Darmstadt, Germany) and treated by sonication (Microson XL‐2000, Minisonix, Farmingdale, NY, USA). Aliquots of supernatants containing equal amounts of protein (50 µg) in Laemmli buffer were separated on Bolt Bis‐Tris Plus 4–12% precast polyacrylamide gels (Life Technologies, Monza, Italy). Fractionated proteins were transferred from the gel to a PVDF nitrocellulose membrane using an iBlot 2 system (Life Technologies, Monza, Italy). Blots were stained with Ponceau red to ensure equal loading and complete transfer of proteins, and then blocked for 1 h at room temperature with 6% milk in PBS containing 0.1% Tween. Subsequently, membranes were probed at 4 °C overnight with GPX4 (Cell Signaling), FTH1 (Cell Signaling), HO‐1 (Cell Signaling), NCOA4 (Cell Signaling), KEAP1 (Cell Signaling), H2AX (Cell Signaling), p62 (Cell Signaling), Beclin‐1 (Cell Signaling), LC3A/B (ThermoFisher), Vinculin (Cell Signaling), GAPDH (Cell Signaling). Antirabbit IgG (whole molecule)–Peroxidase antibody (Sigma, Cat#A0545) or antimouse IgG (whole molecule)–Peroxidase antibody (Sigma, Cat#A9044) were used as secondary antibodies; the enhanced chemiluminescence (ECL) procedure was employed for development.

### Formalin‐Fixed Paraffin‐Embedded (FFPE) RNA Extraction Sample and Quantitative RT‐PCR (qRT‐PCR)

Twelve 10 µm thick sections were cut from each block of FFPE tissue, transferred to 1.5 mL sterile tubes, and processed using the PureLink FFPE Total RNA Isolation Kit (Invitrogen, Thermofisher) according to manufacturer's protocol. RNA was extracted by spin column purification according to similar basic principles: deparaffinization, followed by cell disruption with heated proteinase K, which is capable of efficiently degrading proteins that were covalently crosslinked with each other and RNA. Proteinase K incubation at high temperature (60 to 70 °C) also removes part of the methylol additions induced by formalin fixation. After proteinase K incubation, RNA was isolated by alcohol precipitation in a spin column purification step and then was stored at −80 °C. Total RNA 260/280 OD ratios were consistently between 1.7 and 1.85, indicating high sample purity. 500 ng RNA was reverse‐transcribed using Thermo Scientific Maxima H Minus cDNA Synthesis Master Mix with dsDNase (Invitrogen, by Thermofisher) according to manufacturer's instructions. Real‐time RT‐PCR was performed using CFX96 Touch Real‐Time PCR Detection System (BioRad, Milano, Italy). The relative expression level of the house‐keeping gene (18S‐rRNA) and three target genes (PCNA, N‐cadherin, and Vimentin) was measured using SYBR Green dye‐based method. Relative mRNA expression of a target gene within a specimen was calculated as 2^‐ ΔC T^ where ΔC T = C T (target gene) − C T (housekeeping gene). The primer sequences were as follows:

Human PCNA: forward TCCTCCTTCCCGCCTGCCTGTAGC and reverse CGCGTTATCTTCGGCCCTTAGTGTA
Human N‐Cadherin Forward CCTCCAGAGTTTACTGCCATGAC and reverse GTAGGATCTCCGCCACTGATTC
Human Vimentin forward AGGCAAAGCAGGAGTCCACTGA and reverse ATCTGGCGTTCCAGGGACTCAT
Human 18S‐rRNA: forward CCAGTAAGTGCGGGTCATAAG and reverse GCCTCACATAA‐CCATCCAATC.

### Radiotherapy Treatment

ECFCs, A375, M6, MCF‐7 and MDA were seeded in T25 flasks at a density of 1 × 10^6^ cells and allowed to reach 70% confluence. Cells were then treated with AuNRs at the concentration 100 × 10^−6^ M Au before being irradiated with Xrays delivered by linear accelerator (Elekta Versa HD, 6 MV) properly set to deliver an exact, homogeneous dose at the level of the cell layers Single doses of 2, 4 and 6 Gy were delivered to the cell cultures. Cells were harvested by trypsin treatment counted with a hemocytometer before plating colony formation assay. Survival rate was then calculated two weeks later. For all further experiments, single dose of 2Gy was delivered to co‐cultures.

### Assay Measurement of DNA Strand Breaks and Oxidized Bases with the Comet Assays

M6 co‐cultured with naive and AuNR‐enriched ECFCs and MCF7 co‐cultured with ECFCs that had undergone radiotherapy, hyperthermia and combo treatment, were aliquoted (50 000 cells in 50 µL) and resuspended in freezing medium (culture medium containing 40% FBS and 10% DMSO) and stored at −80 °C. The frozen samples were de‐frosted and analyzed for DNA damage by Comet assay as previously described.^[^ ^87^ ^]^ Briefly, each aliquot was rapidly melted in a 37 °C water bath and immediately added with 167 µL of melted LMP agarose (LMA, 1% in PBS) kept at 37 °C, to obtain a final 0.7% LMA concentration. Aliquots (70 µL) of this suspension were transferred onto clear microscopy slides pre‐coated with agarose and covered with 20 × 20 mm coverslips (2 duplicate gels per slide). Cells were lysed at 4 °C for 1 h (lysis solution: NaCl 2.5 M, Na2EDTA 100 mM, Tris‐HCl 10 mM, TritonX‐100 1%, pH 10). The obtained nucleoids were then subjected to an alkaline unwinding step by incubating the slides for 20 min at 4 °C in alkaline electrophoresis buffer (NaOH 300 mM, Na2EDTA 1 mM, pH 13). Following electrophoresis, slides were neutralized with two washes in 0.4 M Tris‐HCl, pH 7.4, briefly washed in distilled water and dried o/n at RT. On the following day, gels were stained with SybrGold (Invitrogen, 1:10000 in Tris‐EDTA buffer) and left two days in the fridge. On the day of the microscopic analysis, images of the nucleoids were acquired and analyzed using the Comet Assay IV image analysis system (Perceptive Instruments) coupled to a Nikon Labophot‐2 epifluorescence microscope. Tail migration values (expressed as % tail DNA) were recorded for 100 randomly chosen cells for each experimental point and averaged. Each experiment was repeated three times, and the corresponding values were further averaged for each experimental point and expressed as mean ± SEM. For oxidatively generated damage detection, slides were washed with Enzyme Buffer (100 mM KCl, 0.5 mM EDTA, 40 mM HEPES, and 0.2 mg L^−1^ BSA) 2 times for 5 min at 4 °C, then incubated with 30 µL FPG enzyme per gel (crude E. Coli extract, Norgenotech, Oslo, Norway) or buffer only, for 45 min at 37 °C. The DNA‐formamidopyrimidine glycosylase (Fpg) enzyme was used at 1:60.000 dilution. After this treatment, all slides were directed to the unwinding step as described above. The net FPG sites, corresponding to the level of oxidized bases, were finally calculated by subtracting the % tail DNA value obtained in slides treated with enzyme buffer from that of the corresponding slides treated with the enzyme.

### Immunofluorescence

Cells were grown on µ‐Slide 8 Well (ibidi), washed twice with 1 mL of PBS, fixed for 20 min in 3.7% paraformaldehyde in PBS, and permeabilized with 0.1% Triton X‐100 in PBS for 5 min. Cells were incubated in blocking buffer (3% BSA and 0.1% Triton X‐100 in PBS) for 1 h at room temperature and then the expression of γH2Ax (Cell Signaling) and 4‐HNE (Invitrogen) was evaluated. The primary antibody incubation was performed o/n at +4 °C. Secondary antibody AlexaFluor488 and AlexaFluor633 (Invitrogen) was incubated 1h at RT. Nuclei were stained with fluorescent DAPI (10 µg mL^−1^) (Invitrogen) for 15 min at RT. The chambers were then washed several times with PBS and observed under TCS SP8 microscope (Leica Microsystems) with LAS‐AF image acquisition software.

### DCFDA Assay and BioTracker NIR 633 Lysosome Dye Assay

Co‐culture of M6 and ECFC cells were grown on µ‐Slide 8 Well (ibidi), washed twice with 300 µL of PBS, then 300 µL of a complete medium that was pre‐equilibrated at 37 °C with 10 µM of DCFDA (Sigma Aldrich). The cells were then incubated for 30 min at 37 °C, in the dark. A positive control sample was prepared adding 20 mM H_2_O_2_ during the incubation with the dye. Co‐culture of MCF‐7 and ECFC cells were grown on µ‐Slide 8 Well (ibidi), washed twice with 300 µL of PBS, then 300 µL of a complete medium that was pre‐equilibrated at 37 °C with BioTracker NIR633 Lysosome Dye (Sigma‐Aldrich) for 30 min at 37 °C, according to manufacturer's indications. The cells were promptly imaged under a TCS SP8 microscope (Leica Microsystems) with LAS‐AF image acquisition software.

### Bodipy

After the indicated treatments, the co‐culture of M6 and ECFC cells were grown on µ‐Slide 8 Well (ibidi). Lipid peroxidation was assessed using BODIPY 581/591 C11 undecanoic acid (ThermoFisher) following manufacturers’ instruction. After fixation, nuclei were stained with DAPI (Sigma). Cells were examined and captured using a TCS SP8 microscope (Leica Microsystem) with LAS‐AF software acquisition. Oxidation of the polyunsaturated butadienyl portion of this fatty acid analog in live cells results in a shift of the fluorescence emission peak from red (∼590 nm) to green (∼510 nm).

### Autophagy Detection

After the indicated treatments, the co‐culture of MCF‐7 and ECFC cells were grown on µ‐Slide 8 Well (ibidi). Autophagy was assessed using the CYTO‐ID Autophagy Detection Kit (Enzo Life Sciences) following the manufacturer's instructions. Autophagosomes were visualized using the CYTO‐ID Green Detection Reagent, while nuclei were stained with Hoechst 33342. The stained cells were examined, and images were captured using TCS SP8 microscope (Leica Microsystems) with LAS‐AF image acquisition software.

### Statistical Analysis

For statistical analysis the data were analysed using GraphPad Prism6 and Origin and expressed as a mean value ±SD. Statistical analysis was performed using One way Anova.

This work was financially supported by Associazione Italiana Ricerca sul Cancro (AIRC) grant IG 2020 N. 24 381, by Tuscany Region (Call on Health Bando Ricerca Salute 2018 through Project “THERMINATOR”) and by NextGenerationEU and Ministry of Enterprises and Made in Italy through Project POCARNO 2022.

Open access publishing facilitated by Universita degli Studi di Firenze, as part of the Wiley ‐ CRUI‐CARE agreement.

## Conflict of Interest

The authors declare no conflict of interest.

## Author Contributions

C.A. and F. S. contributed equally to this work. A.L., F.R. conceived, designed the study. C.A, F.S., A.C. S.B. performed and designed the animal experiments and the cytological analysis. F.R, C.B., F.M. synthesized and characterized AuNPs. F.M., E.F, C.A., N.F. and performed molecular analysis. J.R. and C.A preformed confocal image analisys. P.N. and D.G. carried out TEM analysis. G.F., M.D.R. and A.L., analyzed and interpreted the data. R.T and M.S. performed the ICP analysis on cells. A.L wrote and revised the manuscript. G‐F. and A.L obtained fundings. L.M., P.A. and C.C designed and performed the photoacoustic imaging experiments in vitro and in vivo. C.T., M.M. and I.D. designed the protocol and performed the X‐ray irradiation tests. F.R, F.M. designed and performed the NIR irradiation experiments. L.G performed the comet assay experiments. F.P. revised manuscript and provided infrastructures.

## Supporting information



Supporting Information

Supplemental Movie 1

## Data Availability

The data that support the findings of this study are available from the corresponding author upon reasonable request.
