# Supplementary material for: Antitumoral Efficacy of AuNRs‐Laden ECFCs In Vitro and In Vivo: Decoding the Heat and Rays Combo Treatment in Breast Cancer and Melanoma Cells
Source: Adv Healthc Mater. 2025 Jun 23;14(22):2502416. doi: 10.1002/adhm.202502416 (PMC12391628; doi:10.1002/adhm.202502416)
Supplement: Supplementary file 1 — Supporting Information [file ADHM-14-0-s001.docx]

**Antitumoral efficacy of AuNRs-laden ECFCs in vitro and in vivo: decoding the Heat and Rays combo treatment in Breast Cancer and Melanoma Cells.**

*C. Anceschi^1*^, F. Scavone^1*^, P. Armanetti^2^, L. Menichetti^2^, C. Catarinicchia^2^, C.Borri^3^. F.Ratto^3^, F.Micheletti^3^, N. Formica^1,^ J. Ruzzolini^1^, E. Frediani^1^, A. Chillà^1^, F. Margheri^1^, M. Severi^4^, R. Traversi^4^, P. Nardini^5^, D. Guasti^5^, M. Del Rosso^1^, T. Del Rosso^6^, L. Giovanelli^7^, C. Talamonti^1^, M. Mangoni^1^, I. Desideri^1^, S. Burchielli^8^ F. Pajar^9^, G. Fibbi^1#^, A. Laurenzana^1#^*

^1^University of Florence; Department of Experimental and Clinical Biomedical Sciences,^1^Viale Morgagni 50, 50134, Florence, Italy

^2^ National Research Council, Institute of Clinical Physiology (IFC), Pisa 56124, Italy

^3^ Institute of Applied Physics “N. Carrara” National Research Council Sesto Fiorentino 50019, Italy

^4^ University of Florence Department of Chemistry "Ugo Schiff" University of Florence Sesto Fiorentino 50019 Italy.

^5^ University of Florence Department of Experimental and Clinical Medicine University of Florence Florence 50134 Italy, Viale Pieraccini 6 50134 Florence

^6^ Pontifical Catholic University of Rio de Janeiro, Department of Physics, Rua Marquês de São Vicente 225, 22451-900 Gávea, Rio de Janeiro, Brazil

^7^ University of Florence Department of Neurosciences, Psychology, Drug and Child Health Area (NEUROFARBA) Viale Pieraccini 6 50134 Florence

^8^ Center for Experimental Biomedicine, Research Area of CNR Pisa

*^9^* University of Pisa, Dep. of Translational Research and New Technologies, St. Chiara Hospital, Via Savi, 10 – 56100,Pisa

* These authors contributed equally to this work

#Correspondance:[anna.laurenzana@unifi.it](mailto:anna.laurenzana@unifi.it)

Gabriella.fibbi@unifi.it


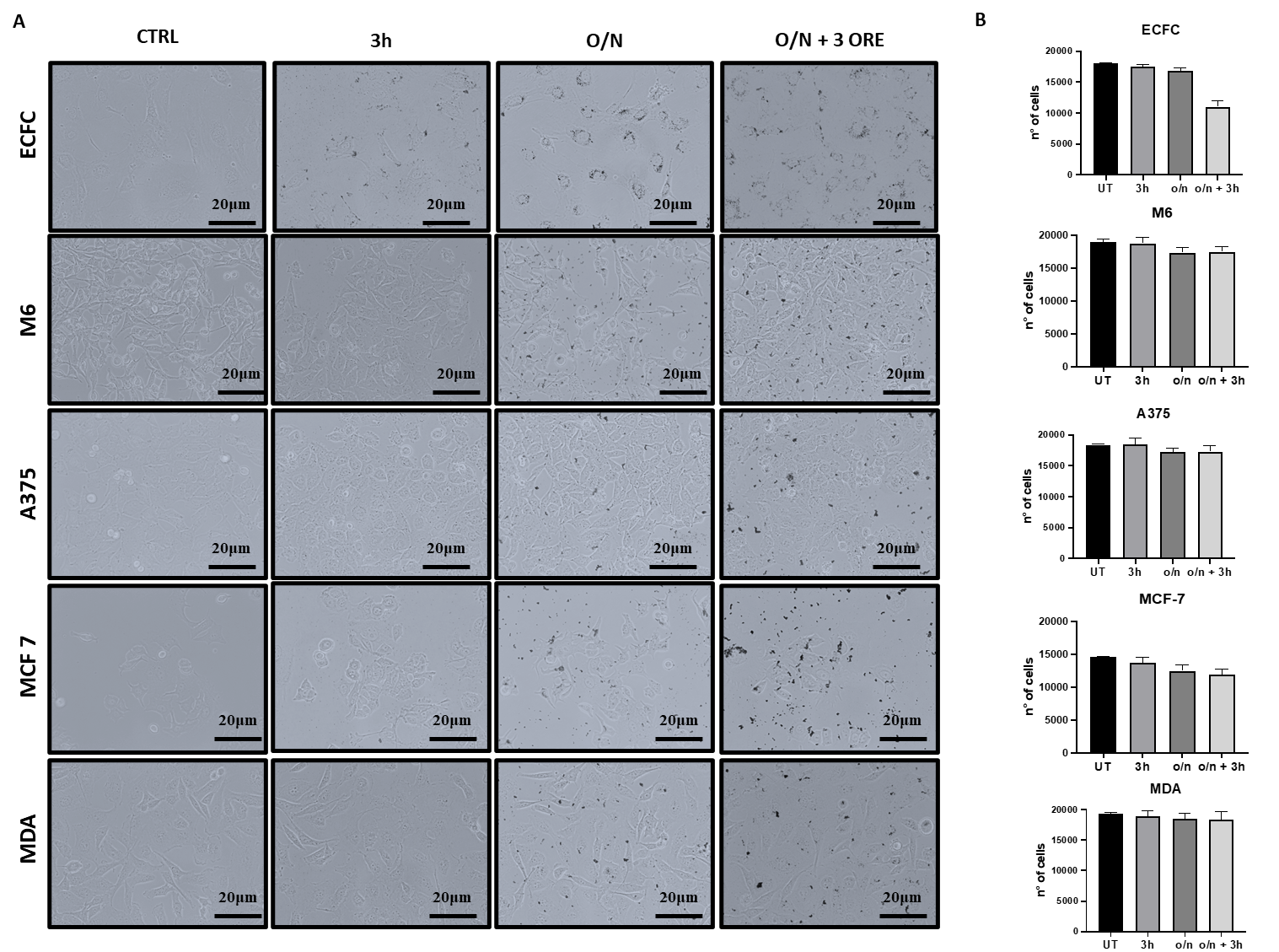


**Figure S1. Time-course uptake of gold nanorods (AuNRs) and corresponding cell viability in ECFCs and various cancer cell lines.** (A) Brightfield optical images show the intracellular uptake of AuNRs in Endothelial Colony Forming Cells (ECFCs), melanoma cell lines (A375 and M6), and breast cancer cell lines (MCF-7 and MDA-MB-231) at different time points (e.g., 3 h, on, and on+3 h). AuNR accumulation is visible as dark intracellular aggregates. (B) Cell viability was quantitatively assessed at each time point using the MTT assay. Data are presented as mean ± SD from three independent experiments.


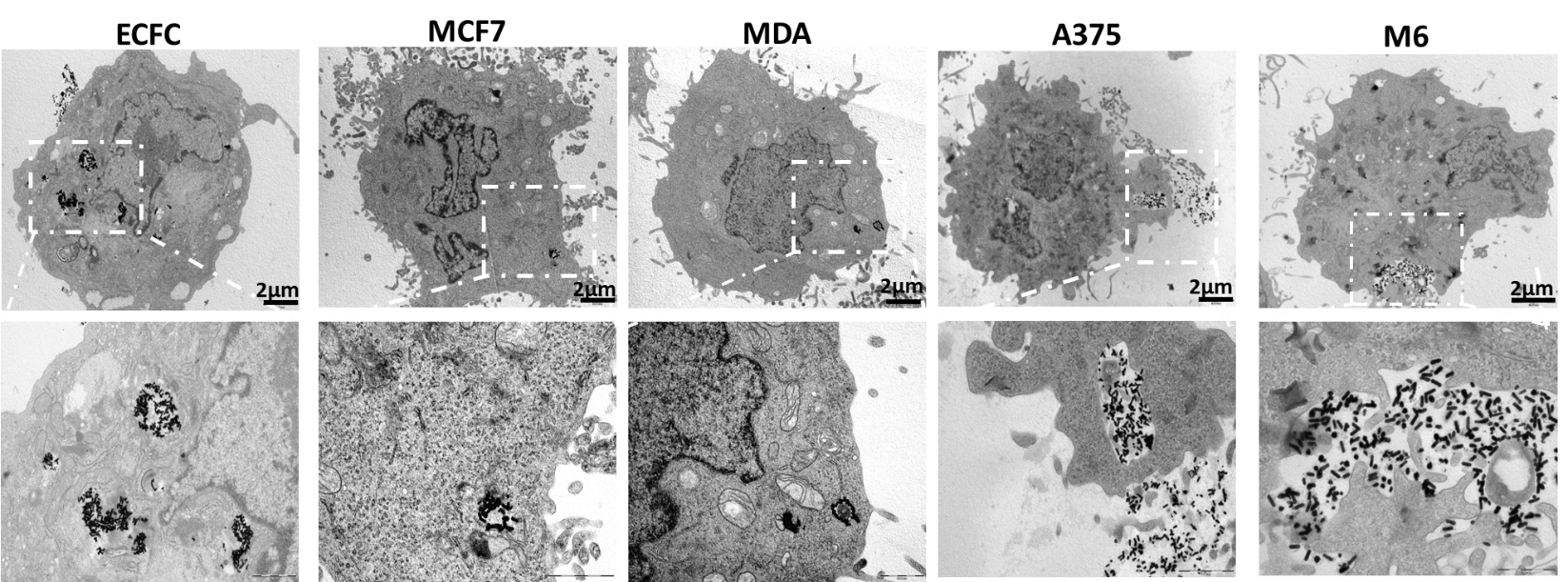


**Figure S2**. **Transmission electron microscopy (TEM) images of ECFCs and cancer cell lines following overnight treatment with AuNRs**. Representative TEM images show the intracellular localization of AuNRs in ECFCs, MCF-7, MDA-MB-231, A375 and M6 cells after overnight (on) exposure. AuNRs appear as electron-dense rod-shaped structures within endosomal compartments and cytoplasmic regions. The bottom panels show higher-magnification views of the selected areas indicated in the corresponding upper panels, highlighting AuNR accumulation and subcellular distribution.


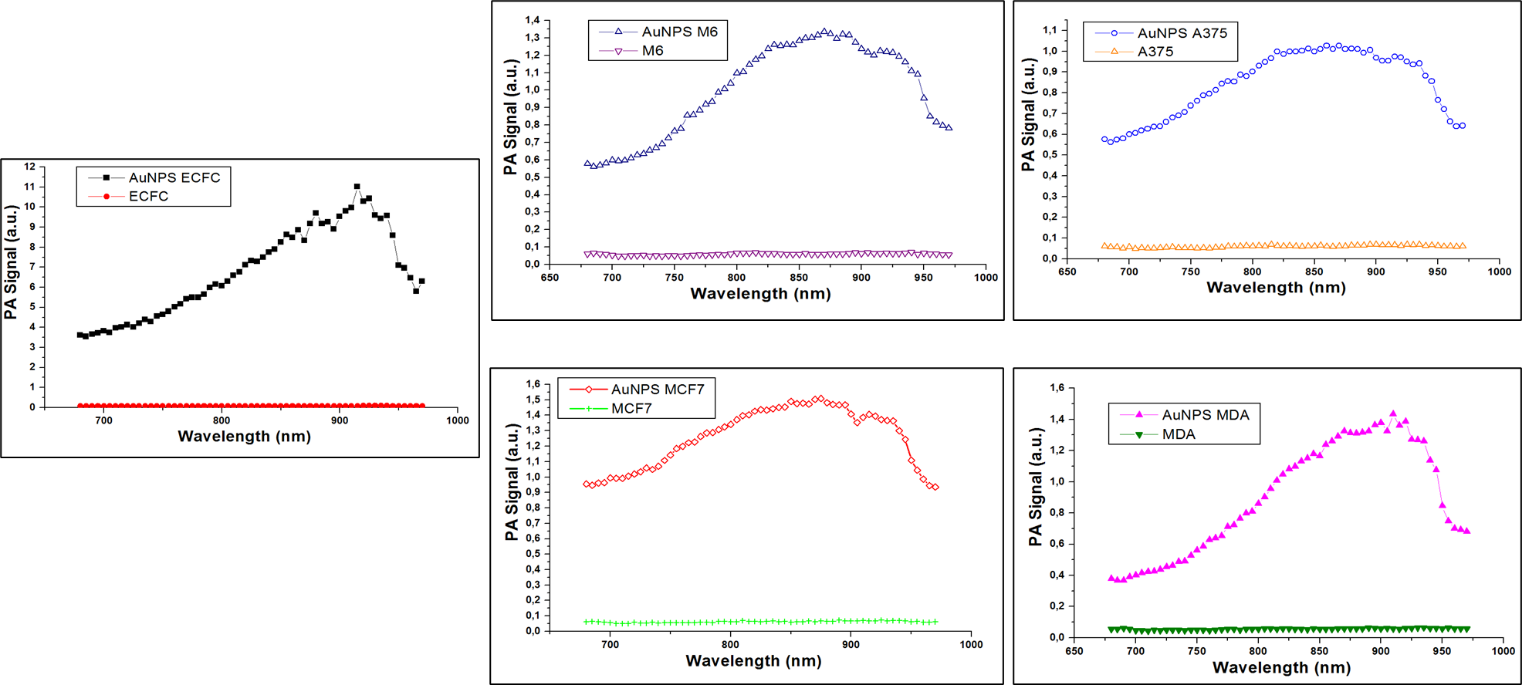


**Figure S3. Photoacoustic spectral (PA) profiles of AuNR-treated ECFCs and cancer cell lines. PA of ECFCs, M6, A375, MCF-7, and MDA-MB-231 cells following treatment with gold nanorods (AuNRs). The plot displays photoacoustic signal intensity as a function of wavelength, highlighting the characteristic spectral signatures of intracellular AuNRs in different cell types. In contrast, untreated control cells did not exhibit any detectable photoacoustic signal across the measured spectral range. Data are representative of three independent experiments.**


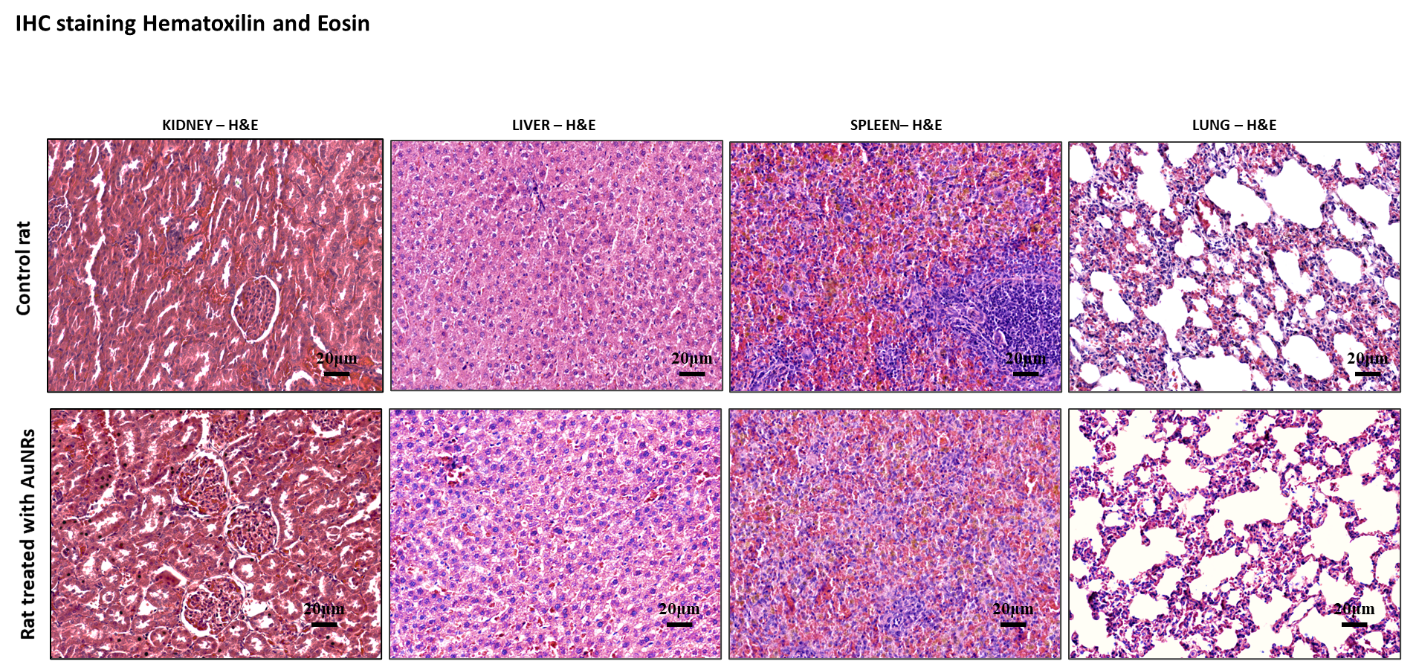


**Figure S4. Histological examination of major organs from AuNR-treated and untreated rats.**
Representative hematoossilin and eosin (H&E-stained sections) of liver, kidney, lung, and spleen collected from rats treated with gold nanorods (AuNRs) and untreated control rats. Microscopic examination was performed to evaluate potential tissue alterations or signs of toxicity. No major histopathological abnormalities were observed in either group, indicating no evident acute toxicity associated with AuNR administration.


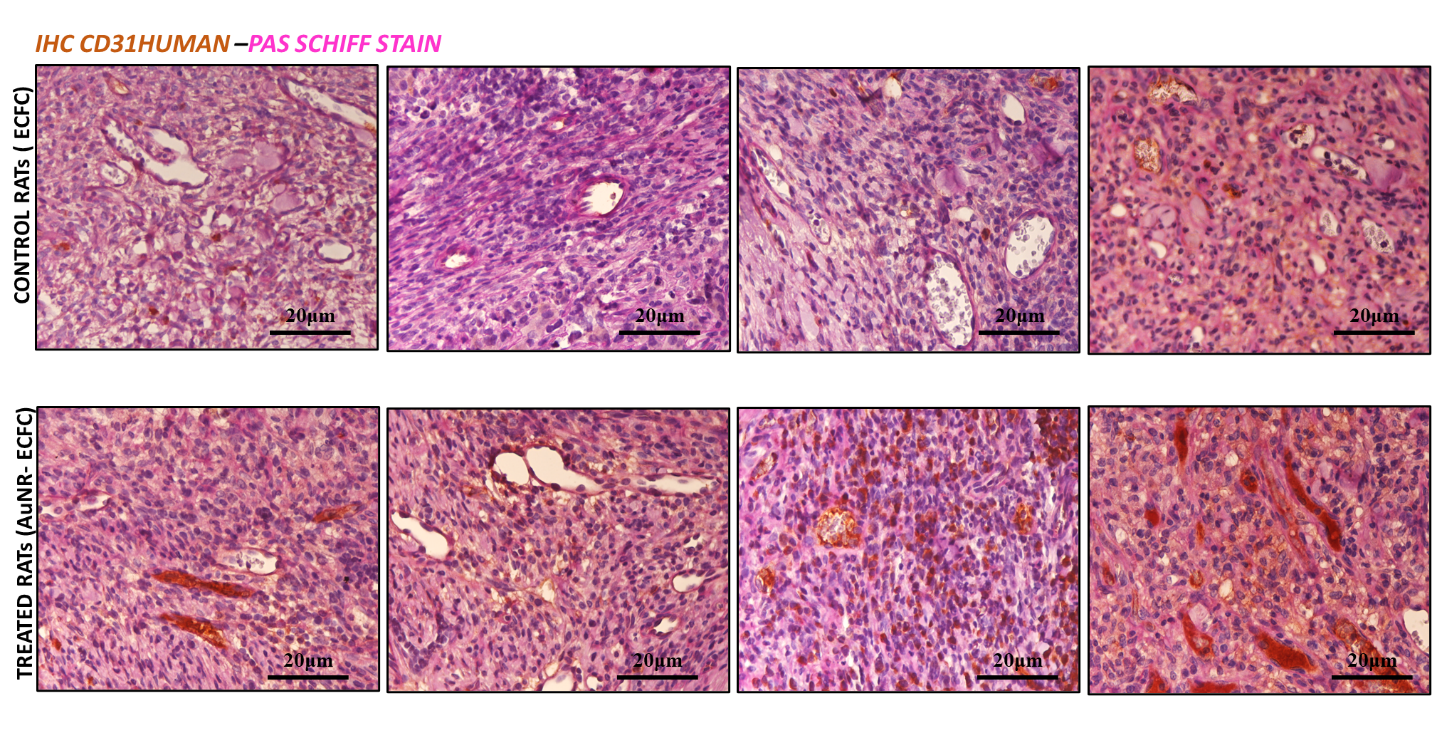


**Figure S5. Representative histological analysis of tumor sections stained for human CD31 and PAS.** Representative images of histological sections from tumor masses in different rats stained with human CD31 (brown) and Periodic Acid-Schiff (PAS; pink). CD31 staining identifies human endothelial cells, while PAS highlights glycogen and other carbohydrate-rich components, including basement membrane-like structures. Regions showing PAS positivity in the absence of CD31 staining are indicative of vascular mimicry—non-endothelial, tumor-derived channels that mimic blood vessels. This dual staining allows discrimination between bona fide endothelial vessels and vascular mimicry structures within the tumor microenvironment.

**A**


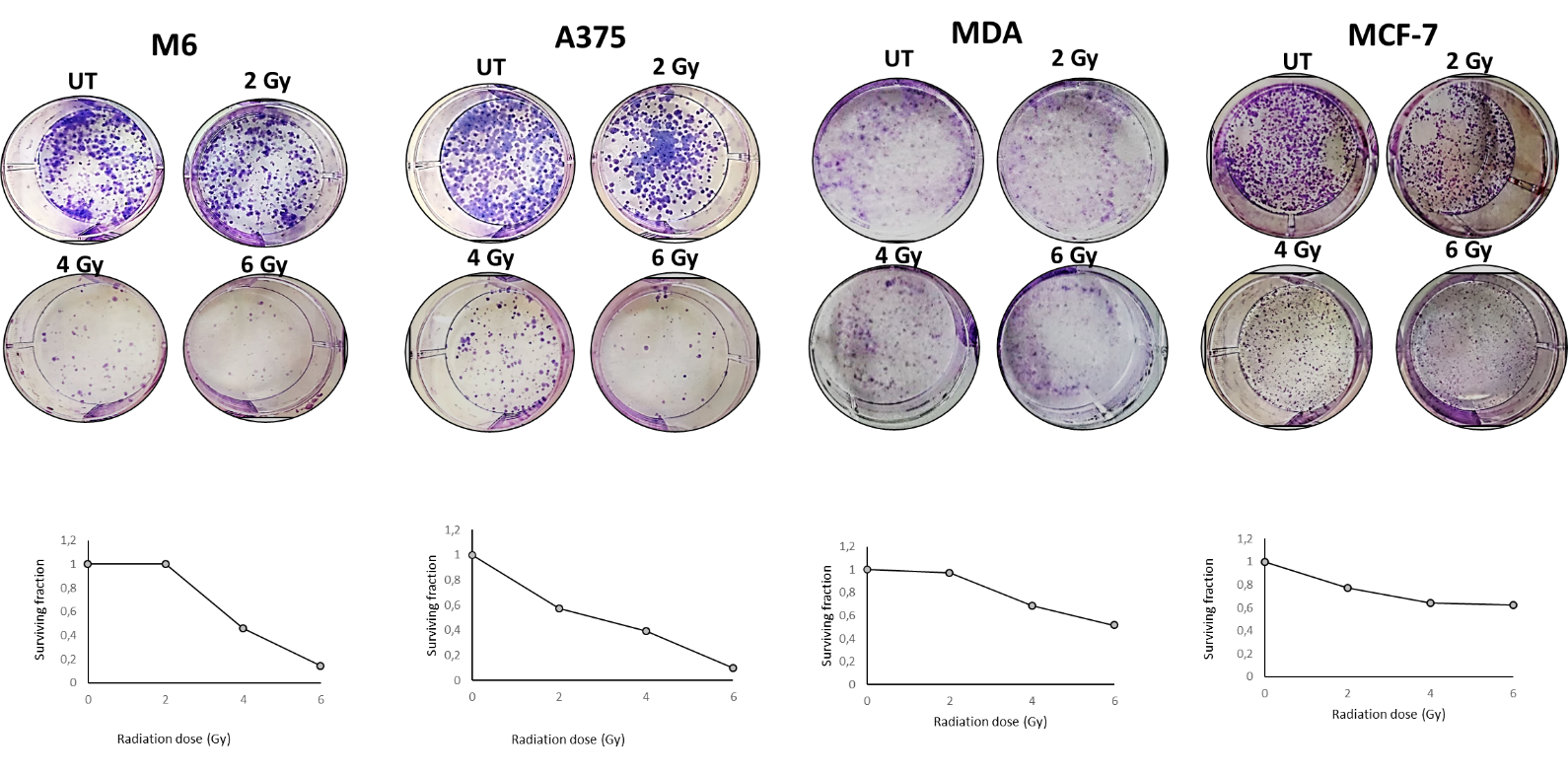


**B**


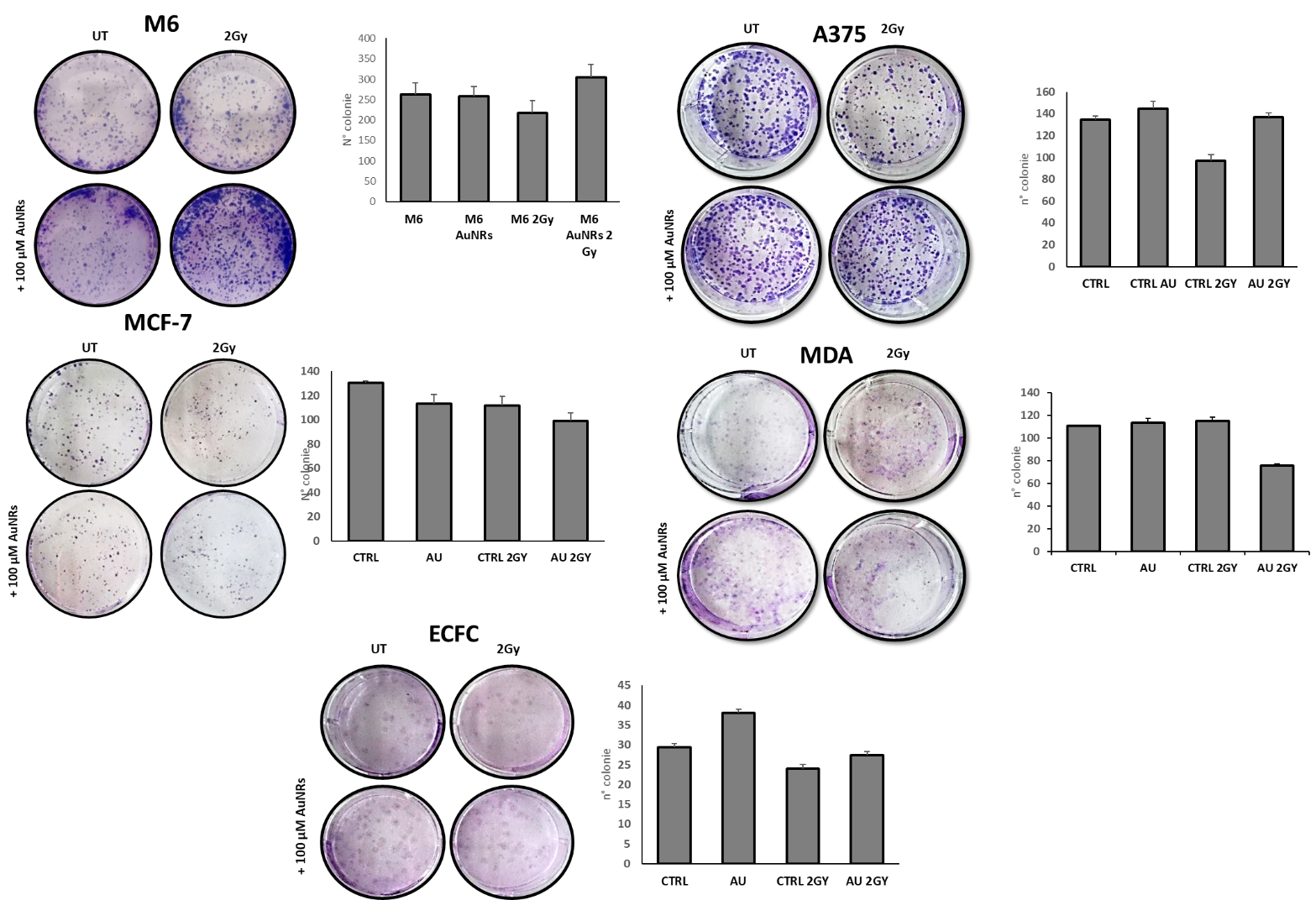


**Figure S6. Clonogenic survival of ECFCs and cancer cell lines following irradiation and AuNR treatment.** **Top panel A:** Clonogenic assay of ECFCs, M6, A375, MDA-MB-231 and MCF-7 cells exposed to increasing doses of ionizing radiation (2–8 Gy). Colony formation efficiency was assessed 10–14 days post-irradiation to evaluate radiosensitivity. **Bottom panel B:** Clonogenic survival of the same cell lines after exposure to 2 Gy irradiation in the presence or absence of gold nanorods (AuNRs). The combination treatment was used to assess potential radiosensitizing effects of AuNRs. Data represent mean ± SD of three independent experiments.

MCF-7 ferroptosis

**2GY**

**43°C**

**2GY+43°C**

**UT**

**- +**

**- +**

**- +**

**- +**

**NCOA4**

**SLC7A11**


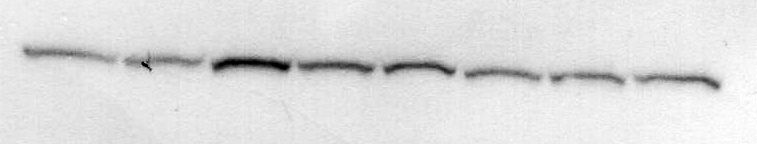

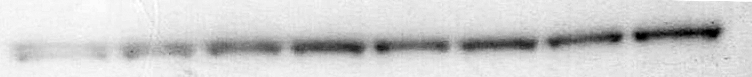

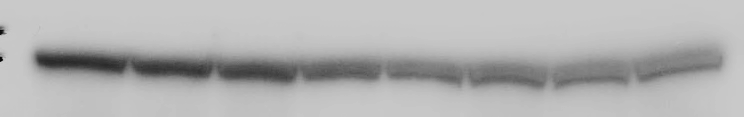


**GPX4**

**P-NRF2**


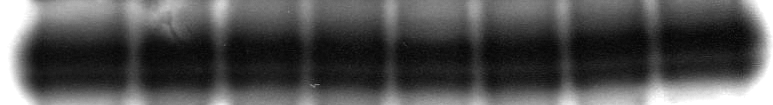

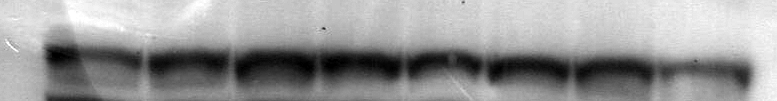


**NRF2**

**FTH1**


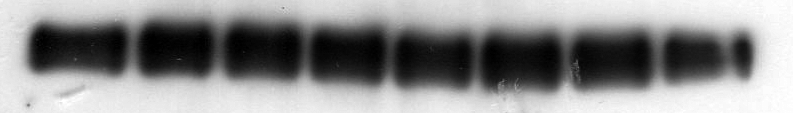

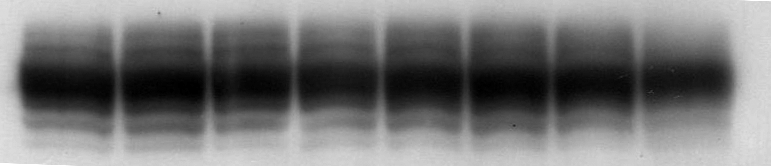


**CD98**


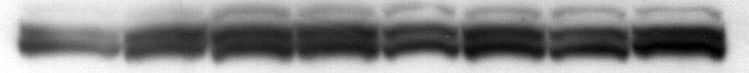


**KEAP1**


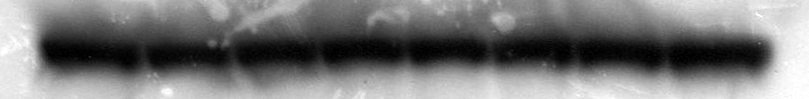


**GADPH**

**Figure S7. Western blot analysis of ferroptosis-related markers in MCF-7 cells following hyperthermia, radiation, and combination treatment.** Western blot analysis was performed to assess the expression of key ferroptosis-related proteins in MCF-7 cells subjected to hyperthermia, ionizing radiation, or their combination. Proteins analyzed included GPX4, SLC7A11, NRF2, FTH1, KEAP1, NCOA4, and CD98, with GAPDH used as a loading control. No significant alterations in the expression levels of these markers were observed under any treatment condition, suggesting that ferroptotic signaling pathways were not notably activated in response to the tested treatments.
